# Introduction of the *Exocelinacasuarina*-group, with a key to its representatives and descriptions of 19 new species from New Guinea (Coleoptera, Dytiscidae, Copelatinae)

**DOI:** 10.3897/zookeys.803.28903

**Published:** 2018-12-06

**Authors:** Helena Shaverdo, Katayo Sagata, Michael Balke

**Affiliations:** 1 Naturhistorisches Museum Wien, Burgring 7, 1010 Vienna, Austria Naturhistorisches Museum Wien Vienna Austria; 2 Papua New Guinea Institute for Biological research (PNG-IBR), Goroka, Papua New Guinea Papua New Guinea Institute for Biological research Goroka Papua New Guinea; 3 SNSB – Zoologische Staatssammlung München, Münchhausenstraße 21, D-81247 Munich, Germany and GeoBioCenter, Ludwig-Maximilians-University, Munich, Germany Zoologische Staatssammlung München Munich Germany

**Keywords:** Copelatinae, Dytiscidae, *Exocelinacasuarina*-group, key, New Guinea, new species

## Abstract

Nineteen new species of *Exocelina* Broun, 1886 from New Guinea are described herein: *E.adelbertensis***sp. n.**, *E.ambua***sp. n.**, *E.bewani***sp. n.**, *E.cyclops***sp. n.**, *E.ibalimi***sp. n.**, *E.keki***sp. n.**, *E.kumulensis***sp. n.**, *E.mendiensis***sp. n.**, *E.menyamya***sp. n.**, *E.okapa***sp. n.**, *E.piusi***sp. n.**, *E.pseudofume***sp. n.**, *E.pseudopusilla***sp. n.**, *E.pusilla***sp. n.**, *E.sima***sp. n.**, *E.simbaiensis***sp. n.**, *E.simbaijimi***sp. n.**, *E.sumokedi***sp. n.**, and *E.yoginofi***sp. n**. All of them, together with five already described species, have been united into the newly defined *casuarina*-group, a polyphyletic complex of related species with similar shape of the median lobe and paramere setation. An identification key to all known species of the group is provided, and important diagnostic characters (habitus, color, male protarsomeres 4–5, median lobes, and parameres) are illustrated. Data on the distribution of the species are given, showing that most of the species occur in the central, mountain part of Papua New Guinea.

## Introduction

**Figure 1.**
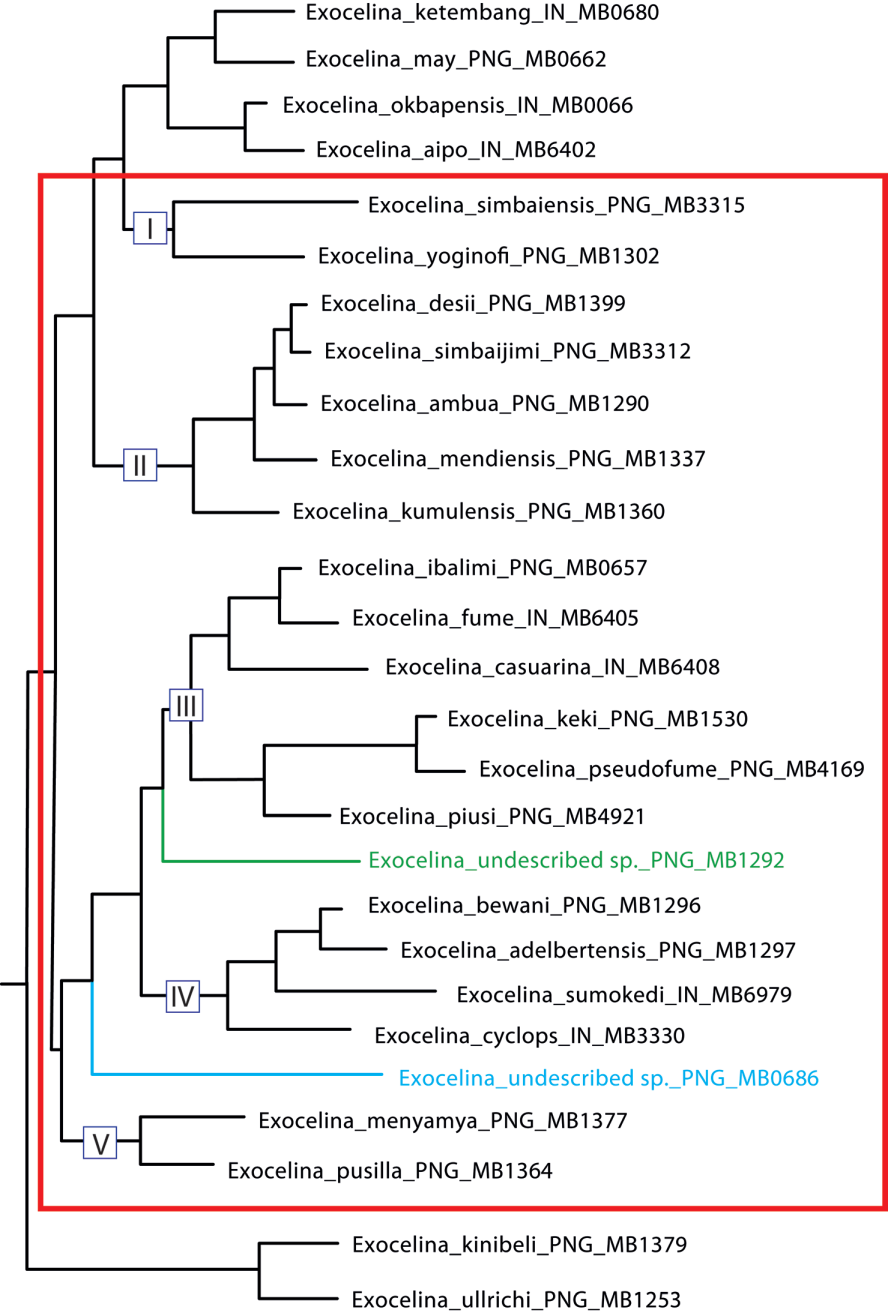
Phylogenetic relationships of the *Exocelinacasuarina*-group based on the MrBayes phylogenetic tree in figure S1 of Toussaint et al. (2015).

Herein, we introduce the new species group of the genus *Exocelina* Broun, 1886. After the *ekari*-group with 51 species, it is the second largest species group of the New Guinea *Exocelina* (Balke et al. 2007, Shaverdo et al. 2012, 2014, 2016a). The group includes 24 species: 19 new species, which are described and illustrated here, and five previously known species: *E.casuarina* (Balke, 1998), *E.fume* (Balke, 1998), *E.desii* (Balke, 1999), *E.heidiae* (Balke, 1998), and *E.messeri* (Balke, 1999). Based on the results of a molecular phylogenetic analysis and morphological study, these species are suggested to be closely related and form a monophyletic complex including two monotypic groups (undescribed species) and the *okbapensis*- and *aipo*-groups (Fig. 1; Balke 1998; Balke et al. 2007; Shaverdo and Balke 2014; Shaverdo et al. 2017; Toussaint et al. 2014, 2015). Also, they build the core of a larger monophyletic complex (including the *ullrichi*-group), which is a sister clade to all other New Guinea *Exocelina* (Toussaint et al. 2015). Morphologically, species of the *casuarina*-group are identified by a complex of characters, among which the important ones are shape of the paramere and median lobe and setation of the paramere.

Including the results of this work, 125 species of *Exocelina* are described from New Guinea and 180 species worldwide.

As in most of our previous papers on the genus (Shaverdo et al. 2012, 2013, 2014, 2016a, b, c, 2017), all species data will be presented on the species-id.net portal automatically created by ZooKeys with the publication of this paper.

## Material and methods

The present work is based on material from the following collections:

**ANIC** Australian National Insect Collection, Canberra, Australia

**BMNH** The Natural History Museum, London, UK

**CLH** Collection of Lars Hendrich, Munich, Germany (property of NHMW)

**IECA** Institute of Entomology, Biology Centre ASCR, České Budějovice, Czech Republic

**MNHN** Muséum National d’Histoire Naturelle, Paris, France

**MZB** Museum Zoologicum Bogoriense, Cibinong, Indonesia

**NHMW** Naturhistorisches Museum Wien, Vienna, Austria

**NHMB** Naturhistorisches Museum Basel, Switzerland

**ZSM** Zoologische Staatsammlung München, Munich, Germany

All methods follow those described in detail in our previous articles (Shaverdo and Balke 2014, Shaverdo et al. 2012, 2014). All specimen data are quoted as they appear on the labels attached to the specimens. Label text is cited using quotation marks. Comments in square brackets are ours. The following abbreviations were used: TL(total body length), TL-H (total body length without head), MW (maximum body width), and hw (handwritten). Figure 1 shows phylogenetic relationships of species within the *Exocelinacasuarina*-group based on the MrBayes phylogenetic tree in figure S1 of Toussaint et al. (2015) and includes results of most recent phylogenetic investigations (Shaverdo and Balke in preparation).

### Checklist and distribution of the species of the *Exocelinacasuarina*-group

Abbreviations: **IN** Indonesia; **PNG** Papua New Guinea.

**Table:** 

	Species	Distribution
1.	*Exocelinaadelbertensis* sp. n.	PNG: Madang
2.	*Exocelinaambua* sp. n.	PNG: Southern Highlands
3.	*Exocelinabewani* sp. n.	PNG: Sandaun
4.	*Exocelinacasuarina* (Balke, 1998)	IN: Papua: Nabire
5.	*Exocelinacyclops* sp. n.	IN: Papua: Jayapura
6.	*Exocelinadesii* (Balke, 1999)	PNG: East Sepik, Simbu, Eastern and Western Highlands
7.	*Exocelinafume* (Balke, 1998)	IN: Papua: Pegunungan Bintang
8.	*Exocelinaheidiae* (Balke, 1998)	PNG: Morobe
9.	*Exocelinaibalimi* sp. n.	PNG: Sandaun
10.	*Exocelinakeki* sp. n.	PNG: Madang, Eastern Highlands
11.	*Exocelinakumulensis* sp. n.	PNG: Enga
12.	*Exocelinamendiensis* sp. n.	PNG: Southern Highlands
13.	*Exocelinamenyamya* sp. n.	PNG: Morobe
14.	*Exocelinamesseri* (Balke, 1999)	PNG: East Sepik
15.	*Exocelinaokapa* sp. n.	PNG: Eastern Highlands
16.	*Exocelinapiusi* sp. n.	PNG: East Sepik
17.	*Exocelinapseudofume* sp. n.	PNG: Madang
18.	*Exocelinapseudopusilla* sp. n.	PNG: Simbu
19.	*Exocelinapusilla* sp. n.	PNG: Madang, Simbu
20.	*Exocelinasima* sp. n.	PNG: Eastern Highlands, Simbu
21.	*Exocelinasimbaiensis* sp. n.	PNG: Western Highlands
22.	*Exocelinasimbaijimi* sp. n.	PNG: Western Highlands
23.	*Exocelinasumokedi* sp. n.	IN: Papua: Puncak
24.	*Exocelinayoginofi* sp. n.	PNG: Eastern Highlands

### Notes on diagnostic characters and phylogeny of the *Exocelinacasuarina*-group

The diagnostic characters of the group are:

– beetles small or medium-sized (TL-H 2.7–5.5 mm);

– habitus elongate to oval, in most species oblong-oval (broadest approximately at elytral midlength); with rounded pronotal and elytral sides, body outline continuous;

– pronotum short, trapezoidal, with posterior angles not drawn backwards;

– coloration reddish to piceous, mainly uniform, sometimes with paler head and pronotum and darker elytra;

– microreticulation and punctation of dorsal surface very fine to strongly impressed, beetles shiny to matt dorsally;

– metacoxae and abdominal ventrites 1–5 (and 6 in males) with thin, almost longitudinal striae/strioles;

– pronotum and elytra without striae or strioles;

– pronotum with or without lateral bead;

– antennomeres not modified;

– male protarsomeres 1–3 not expanded laterally;

– male protarsomere 4 cylindrical, narrow, with anterior angle slightly expanded in some species, with a large, hook-like to thin, long, slightly curved anterolateral seta;

– male protarsomere 5 long and narrow, sometimes slightly concave ventrally;

– median lobe of aedeagus with continuous outline in ventral and lateral view; almost straight or slightly curved in lateral view; in ventral view, almost parallel-sided, often narrowed distally before apex or towards it, or broadened subdistally; apex usually with thickened sides, slightly or distinctly enlarged (“swollen”, often ventrally of shape of a baby pacifier), rounded, truncate, or slightly concave in ventral view;

– ventral sclerite of median lobe more or less deeply divided apically;

– median lobe without setation, in some species with minuscule spines;

– paramere without dorsal notch and with long, dense, thin setae, situated along dorsal margin, subdistal setae usually denser and stronger than proximal ones.

Although the species of the group do not form a monophyletic complex with the distinguished autapomorphic morphological character (Fig. 1; Toussaint et al. 2014, 2015), we designate this species group since its representatives are assumed to be closely related and for ease of their identification. The group can be clearly differentiated (keyed out) using the characters proposed above (also in Shaverdo and Balke in preparation). Additionally, most of its species are readily distinguished by the thickened apex of their median lobe in lateral view, which is a character in common for all the members of this group. Surprisingly, in ventral view, this “swollen” apex can be very differently formed, from broadly pointed to truncate or slightly concave. In addition, the shape (absence of dorsal notch) and setation (subdistal setae denser than proximal ones) of the paramere is useful for species differentiation of this group, especially the few species that do not have this characteristic “swollen” apex or where it is not strongly enough expressed, from some species of the *okbapensis*- and *ransikiensis*-groups.

Phylogenetically, the group is polyphyletic and includes five different clades, which are partially supported morphologically and contribute to two larger monophyletic complexes: 1) clades I and II plus the *okbapensis*-group and 2) clades III, IV, and V plus two monotypic groups (two undescribed species, which are very different morphologically from all the other species of the clades) (Fig. 1; Toussaint et al. 2015).

Clade I includes *E.simbaiensis* sp. n., *E.yoginofi* sp. n. and, probably, *E.okapa* sp. n. (based on morphology). These species build a monophyletic complex with the species of the *okbapensis*- and *aipo*-groups. Interestingly, the two latter species demonstrate a distinct similarity with the species of the *okbapensis*-group in the shape of the median lobe and setation of paramere, though *E.simbaiensis* sp. n. does not.

Clade II is morphologically rather heterogeneous and is comprised of the largest (size) representatives of the group. Three of them, *E.desii*, *E.simbaijimi* sp. n. and, probably, *E.heidiae* (based on morphology), form a monophyletic complex and have broad, similar in shape median lobes. The remainder have median lobes distinctly narrower and more different in shape.

Clade III includes species without lateral pronotal bead, except for *Exocelinapiusi* sp. n., which has narrow but distinct pronotal bead and seems to form a separate lineage. There are two monophyletic complexes in the clade: 1) *E.casuarina*, *E.fume*, and *E.ibalimi* sp. n. with a large, hook-like anterolateral seta of the male protarsomere 4 and 2) *E.keki* sp. n. and *E.pseudofume* sp. n. with a thin, long, slightly curved anterolateral seta of the male protarsomere 4, as well as *E.messeri* and *E.sima* sp. n., which also have a similar shape of this seta. The representatives of this clade best demonstrate a “swollen” apex of the median lobe.

Clade IV is the most homogeneous and includes the smallest in size species of the group. They are morphologically very similar, and three of them, *E.cyclops* sp. n., *E.bewani* sp. n., and *E.adelbertensis* sp. n., are a good example of recent allopatric speciation along the north coast of New Guinea (Toussaint et al. 2014).

Clade V includes two very different species. *Exocelinamenyamya* sp. n. is the most uncharacteristic representative of the group because the apex of its median lobe is thin, flattened, and with ventral impression. The second species is *E.pusilla* sp. n., one of the smallest species of the group. Based on its size and coloration, this species could have been placed into the clade IV but the molecular analysis, as well as the shape of its median lobe, showed that it is a separate lineage of inland mountain *Exocelina*. Most likely *E.pseudopusilla* sp. n. belongs to this clade too. This species is very similar to *E.pusilla* sp. n., but larger and more elongate, with denser and coarser dorsal punctation and microreticulation and different shape of the median lobe (for more details on species delimitation, see the species descriptions). *Exocelinapusilla* sp. n. has wider distribution. Both species are known from the Mount Wilhelm, but from different altitudes: *E.pusilla* sp. n. only from 200 m (from other localities, it is known from up to 500 m) and *E.pseudopusilla* sp. n. only from 1200 m. If this species delimitation is correct, then this is the first distinct example in *Exocelina* of altitudinal peripatric speciation, which is also assumed for the *Exocelina* species of Weyland area (Toussaint et al. 2014).

Thus, this group, as defined now, is the subject of further study and may be divided into subgroups or even groups as and when additional species are discovered.

## Species descriptions (in alphabetic order)

### 1. Exocelina adelbertensis

Taxon classificationAnimaliaColeopteraDytiscidae

Shaverdo & Balke sp. n.

http://zoobank.org/3DB2B98C-7582-4CA5-92CC-A3962AC09EC4

Figs 14 , 38

Exocelina undescribed sp. MB1297: Toussaint et al. 2014: supplementary figs 1–4, table 2;
Toussaint et al. 2015: supplementary figs S1–S2, table S3.
Exocelina adalbert _New_Guinea_MB1297: Toussaint et al. 2015: supplementary information S5–S6.

#### Type locality.

Papua New Guinea: Madang Province, Adelbert Mts, Keki to Sewan, 04°41.80'S, 145.25.46'E, 650 m a.s.l.

#### Type material.

*Holotype*: male “Papua New Guinea: Madang, Adalbert [sic!] Mts., Keki to Sewan, 650m, 7.v.1994, 04.41.802S 145.25.460E, Balke (PNG 54)”, “M.Balke 1297” [green] (ZSM).

#### Description.

*Body size and form*: Beetle small: TL-H 3.1 mm, TL 3.4 mm, MW 1.9 mm, with broader, oval habitus.

*Coloration*: Brownish, with head and pronotum paler. Head reddish brown, darker posterior to eyes. Pronotum reddish brown on sides, dark brown on disc. Elytra brown. Head appendages yellowish red, legs reddish, distally darker, especially metathoracic legs (Fig. 14).

*Surface sculpture*: Submatt dorsally. As in *E.sumokedi* sp. n., except for more strongly impressed microreticulation.

*Structures*: Pronotum with lateral bead. Its lateral sides with longitudinal impressions. Base of prosternum and neck of prosternal process with distinct ridge, rounded anteriorly. Blade of prosternal process lanceolate, relatively broad, slightly convex, with distinct lateral bead and few setae. Abdominal ventrite 6 slightly truncate.

*Male*: Antennae simple (Fig. 14). Protarsomere 4 with medium-sized, thick, curved anterolateral hook-like seta. Protarsomere 5 long and narrow, with anterior row of 17 and posterior row of 6 relatively short, thin setae (Fig. 38D). Median lobe in lateral view slightly curved, with thickened, angulate apex; in ventral view, subparallel, very slightly narrowed distally, and with broadly truncate apex. Paramere slightly concave on dorsal side and with dense setae on subdistal part; proximal setae sparser (Fig. 38A–C). Abdominal ventrite 6 with 4–5 lateral striae on each side.

*Female*: Unknown.

#### Affinities.

*Exocelinaadelbertensis* sp. n. is very similar to *E.sumokedi* sp. n. and *E.bewani* sp. n. but it has slightly more strongly impressed microreticulation, therefore, dorsal surface is distinctly less shiny. Median lobe is more thickened, similar to that of *E.sumokedi* sp. n. but its apex is curved downwards and with stronger terminal angulation. The species is also similar to *E.cyclops* sp. n., *E.pseudopusilla* sp. n., and *E.pusilla* sp. n., see their “Affinities” and the “Key”.

#### Distribution.

Papua New Guinea: Madang Province. The species is known only from the type locality (Fig. 50).

#### Etymology.

The species is named after Adelbert Mountains. The species name is an adjective in the nominative singular.

### 2. Exocelina ambua

Taxon classificationAnimaliaColeopteraDytiscidae

Shaverdo & Balke sp. n.

http://zoobank.org/E9D7093B-F927-45D4-8A84-9E76A639FD39

Figs 21 , 45

Exocelina undescribed sp. MB1290: Toussaint et al. 2014: supplementary figs 1–4, table 2; Toussaint et al. 2015: supplementary figs S1–S2, table S3.
Exocelina ambuaensis _New_Guinea_MB1290: Toussaint et al. 2015: supplementary information S5–S6.

#### Type locality.

Papua New Guinea: Southern Highlands Province, Tari, Mt Ambua, 05°57.55'S, 143°04.99'E, 2,100 m a.s.l.

#### Type material.

*Holotype*: male “Papua New Guinea: Southern Highlands, Tari, Mt Ambua, 2100m, 14.v.2006, 05.57.550S 143.04.993E, Balke (PNG 64)” (ZSM). *Paratypes*: 3 males, 2 females with the same label as the holotype, one of the males with an additional green label “M.Balke 1290” (NHMW, ZSM).

#### Description.

*Body size and form*: Beetle medium-sized: TL-H 4.3–4.7 mm, TL 4.8–5.2 mm, MW 2.2–2.5 mm (holotype: TL-H 4.4 mm, TL 4.8 mm, MW 2.3 mm), with oblong habitus.

*Coloration*: Brown to piceous, with head and pronotum paler. Head reddish brown to piceous, with small darker areas posterior to eyes. Pronotum dark brown to piceous, paler on sides and darker on disc. Elytra dark brown to piceous, with vague narrow reddish to brownish sutural lines. Head appendages and legs proximally reddish brown, legs distally darker, brownish, especially metathoracic legs (Fig. 21).

*Surface sculpture*: Matt dorsally. Head with dense, coarse punctation (no spaces between punctures or spaces 2 times size of punctures), finer and sparser anteriorly; diameter of punctures equal to diameter of cells of microreticulation. Pronotum and elytra with dense, coarse punctation, sparser and finer than on head. Pronotum and elytra with strongly impressed microreticulation. Head with microreticulation stronger. Metaventrite and metacoxae distinctly microreticulate, metacoxal plates with longitudinal strioles and transverse wrinkles, abdominal ventrites with distinct microreticulation and strioles. Metaventrite medially, metacoxal plates, and abdominal ventrites with fine, sparse punctation.

*Structures*: Pronotum with distinct lateral bead. Its lateral sides with distinct longitudinal impressions. Base of prosternum and neck of prosternal process with distinct ridge, rounded anteriorly. Blade of prosternal process lanceolate, relatively narrow, slightly convex, and smooth, with distinct lateral bead and few lateral setae. Abdominal ventrite 6 slightly truncate.

*Male*: Antennae simple (Fig. 21). Protarsomere 4 with anterior angle slightly expanded, with large, thick, strongly curved anterolateral hook-like seta. Protarsomere 5 slightly concave ventrally, with anterior band of ca 70 and posterior band of ca 30 relatively long setae (Fig. 45D). Median lobe in lateral view short, slightly curved, and evenly tapering to dully pointed apex, apex not bent downwards; in ventral view, almost subparallel and distally slightly narrowed to apex, apex roundly truncate. Paramere slightly concave on dorsal side, with long, dense subdistal setae, proximal ones finer (Fig. 45A–C). Abdominal ventrite 6 with 13–15 lateral striae on each side.

*Female*: Without evident differences in external morphology from males, except for not modified protarsi and abdominal ventrite 6 without striae.

#### Affinities.

*Exocelinaambua* sp. n. is similar to *E.mendiensis* sp. n. but differs from it in smaller size, coarser and denser dorsal punctation and microreticulation, and shape of the median lobe.

#### Distribution.

Papua New Guinea: Southern Highlands Province. The species is known only from the type locality (Fig. 50).

#### Etymology.

The species is named after Mt Ambua. The name is a noun in the nominative singular standing in apposition.

### 3. Exocelina bewani

Taxon classificationAnimaliaColeopteraDytiscidae

Shaverdo & Balke sp. n.

http://zoobank.org/8B3BBF57-4E24-446C-BC66-44AB12E15B7E

Figs 13 , 37

Exocelina undescribed sp. MB1296: Toussaint et al. 2014: supplementary figs 1–4, table 2; Toussaint et al. 2015: supplementary figs S1–S2, table S3.
Exocelina bewani _New_Guinea_MB1296: Toussaint et al. 2015: supplementary information S5–S6.

#### Type locality.

Papua New Guinea: Sandaun Province, Bewani Mts, approximately 03°05.13'S, 141°10.23'E., 400 m a.s.l.

#### Type material.

*Holotype*: male “Papua New Guinea: Sandaun, Bewani Stn., limestone creek@base of Bewani Mts., 400 m, 12.iv.2006, nr. 03.05.130S 141.10.227E, Balke & Sagata (PNG 39)”, “M.Balke 1296” [green] (ZSM). *Paratypes*: 2 males, 1 female with the same label as the holotype (NHMW, ZSM).

#### Description.

*Body size and form*: Beetle small: TL-H 3.05–3.25 mm, TL 3.35–3.55 mm, MW 1.8–1.9 mm (holotype: TL-H 3.05 mm, TL 3.35 mm, MW 1.8 mm), with broader, oval habitus.

*Coloration*: Brownish, with head and pronotum paler. Head reddish brown to brownish, sometimes paler anteriorly. Pronotum reddish brown on sides, brown to dark brown on disc. Elytra brown to dark brown, sometimes with narrow reddish sutural lines. Head appendages yellowish red, legs reddish, distally darker, especially metathoracic legs (Fig. 13).

*Surface sculpture*: Shiny but with dense, distinct punctation dorsally. As in *E.sumokedi* sp. n.

*Structures*: Pronotum with lateral bead. Its lateral sides with shallow longitudinal impressions. Base of prosternum and neck of prosternal process with distinct ridge, narrowly rounded anteriorly. Blade of prosternal process lanceolate, relatively broad, slightly convex, with distinct lateral bead and few setae. Abdominal ventrite 6 slightly truncate.

*Male*: Antennae simple (Fig. 13). Protarsomere 4 with medium-sized, thick, curved anterolateral hook-like seta. Protarsomere 5 long and narrow, with anterior row of 21 and posterior row of 4 relatively short setae (Fig. 37D). Median lobe in lateral view slightly curved, with slightly thickened, angulate, and curved downwards apex; in ventral view, subparallel, with broad and slightly concave apex. Paramere slightly concave on dorsal side and with dense setae on subdistal part; proximal setae sparser (Fig. 37A–C). Abdominal ventrite 6 with 3 or 4 lateral striae on each side.

*Female*: Without evident differences in external morphology from males, except for not modified protarsi and abdominal ventrite 6 without striae.

#### Affinities.

*Exocelinabewani* sp. n. is very similar to *E.sumokedi* sp. n. but is larger and has a less striated abdominal ventrite 6; also, its median lobe is not narrowed distally in ventral view and with apex broad and slightly concave ventrally and curved downwards in lateral view. The species is also similar to *E.adelbertensis* sp. n., *E.cyclops* sp. n., *E.pseudopusilla* sp. n., and *E.pusilla* sp. n., see their “Affinities” and the “Key”.

#### Distribution.

Papua New Guinea: Sandaun Province. The species is known only from the type locality (Fig. 50).

#### Etymology.

The species is named after the Bewani Mountains. The name is a noun in the nominative singular standing in apposition.

### 4. Exocelina casuarina

Taxon classificationAnimaliaColeopteraDytiscidae

(Balke & Hendrich, 1998)

Figs 2 , 26

Copelatus (Papuadytes) casuarinus Balke & Hendrich, 1998 in Balke 1998: 328; Nilsson 2001: 76 (catalogue).
Papuadytes casuarinus (Balke & Hendrich, 1998): Nilsson and Fery 2006: 56 (comb. n.).
Exocelina casuarina (Balke & Hendrich, 1998): Nilsson 2007: 33 (comb. n.); Nilsson and Hájek 2018: 65 (catalogue).

#### Type locality.

Papua: Nabire Regency, 62 km of road Nabire to Enarotali, ca 03°30.936'S, 135°42.945'E, 250 m a.s.l. Note: the road only goes up to Enarotali, Ilaga is much further in the mountains, therefore, people now refer to the road as Nabire-Enarotali.

#### Type material studied.

*Holotype*: male “IR 23-W. New Guinea, track Nabire-Ilaga, KM 62, 250m, 24.vii.1991 Balke & Hendrich leg.”, “HOLOTYPUS” [red], “Copelatuscasuarinus Balke des. 1997” [red] (NHMW). *Paratypes*: 3 males with the same labels as the holotype and with red labels “Paratypus Copelatuscasuarinus Balke des. 1997”, one of them additionally with labels “M.Balke 3281” [green] and “M.Balke 6408” [green text] (NHMW).

#### Additional material.

1 female “IRIAN JAYA: Paniai Prov. road Nabire-Ilaga, km 65 29.8.1996, 250m leg. M. Balke (96 # 6)” (NHMW). 4 males, 2 females “West New Guinea/Paniai Prov/JR 22 track Nabire-Ilaga km 62 250m, 24.7.1991, forest pools leg. Balke & Hendrich” (CLH). 1 male “W.-Neuguinea/Paniai Prov. Straße Nabire-Ilaga km 5 700m, 22.-2.9.1990/IR 11 leg: Balke & Hendrich” (CLH).

#### Diagnosis.

**Figures 2–4.**
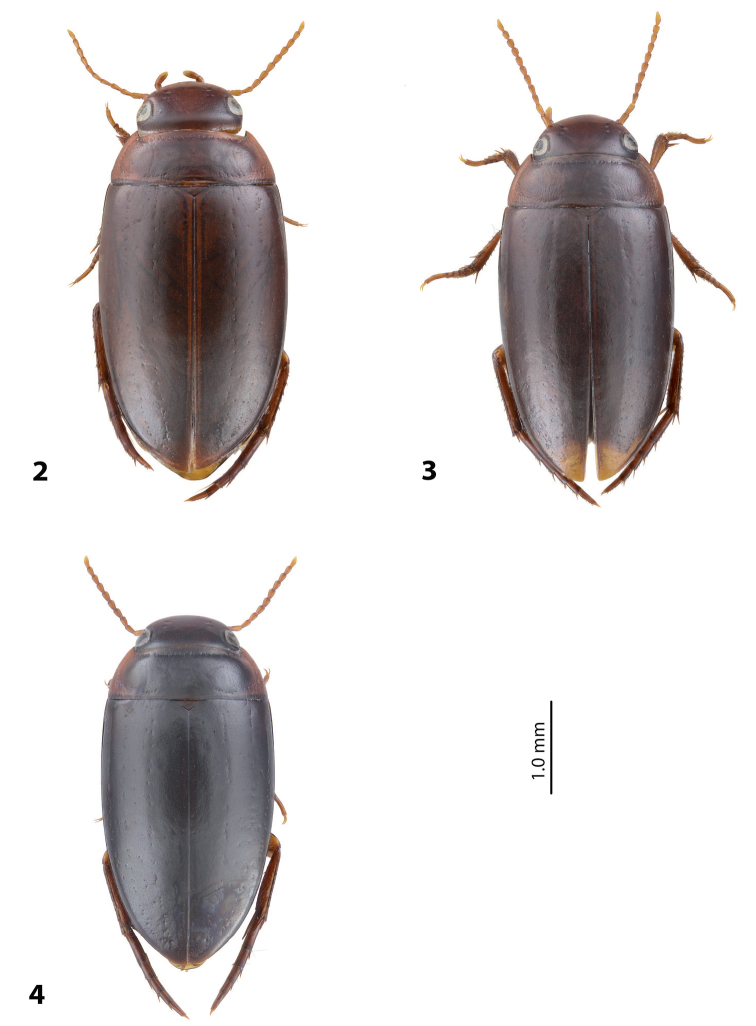
Habitus and coloration **2***Exocelinacasuarina* (Balke, 1998) **3***E.fume* (Balke, 1998) **4***E.ibalimi* sp. n.

For complete description, see Balke (1998: 328). Beetle medium-sized: TL-H 3.6–4.05 mm; oblong-oval; reddish brown to dark brown, sometimes with reddish to reddish brown pronotal sides and head anteriorly; submatt, with fine but rather dense punctation and strongly impressed microreticulation; pronotum without lateral bead; male antennae simple (Fig. 2); male protarsomere 4 with large, thick, strongly curved anterolateral hook-like seta; male protarsomere 5 long and narrow, with anterior band of more than 60 and posterior row of 12 relatively long, thin setae (Fig. 26D); median lobe in lateral view slightly curved and apically rounded, in ventral view, almost subparallel and not narrowed before truncate or slightly concave apex; paramere slightly concave on dorsal side and with long, dense, thin setae, situated along dorsal margin: subdistal setae strong and dense, setae in middle part shorter and sparser, proximal setae long but sparser than subdistal ones (Fig. 26A–C). Female without evident differences in external morphology from males, except for non-modified pro- and mesotarsi and abdominal ventrite 6 without striae.

#### Affinities.

*Exocelinacasuarina* is the only species of the *casuarina*-group in Nabire Regency. In this area, *Exocelina* is represented mainly by the species of the *ekari*-group, which are small in size and have no pronotal bead. From them, as well as from *E.ransikiensis*Shaverdo et al., 2016d with the same characters, the species differs in larger size and the different shape of the median lobe. From *E.bagus* ((Balke & Hendrich, 2001), in Balke (2001)), which is similar in size and surface sculpture to *E.casuarina*, the species differs in simple male antennae and the different shape of the median lobe. From *E.damantiensis* (Balke, 1998) of the *danae*-group, the only species with the pronotal bead in the Nabire-Enarotali area, *E.casuarina* differs in absence of the pronotal bead, evidently stronger dorsal punctation and microreticulation, and the different shape of the median lobe.

Within the *casuarina*-group, the species is more similar to *E.fume* (Balke, 1998) and *E.ibalimi* sp. n., with which it shares not only absence of the pronotal bead, but also a large, strongly curved anterolateral hook-like seta of the male protarsomere 4 (see their “Affinities” and the “Key”).

#### Distribution.

Papua: Nabire Regency. The species is known only from the area close to the type locality (Fig. 50).

### 5. Exocelina cyclops

Taxon classificationAnimaliaColeopteraDytiscidae

Shaverdo & Balke sp. n.

http://zoobank.org/236B93F4-6599-499D-8325-5FB446714AF2

Figs 12 , 36

Exocelina undescribed sp. MB3330: Toussaint et al. 2014: supplementary figs 1–4, table 2; Toussaint et al. 2015: supplementary figs S1–S2, table S3.
Exocelina cyclops New Guinea MB3330: Toussaint et al. 2015: supplementary information S5–S6.

#### Type locality.

Papua: Jayapura Regency, Cyclops Mts, 02°32.03'S, 140°30.41'E, 710 m a.s.l.

#### Type material.

*Holotype*: male “Indonesia: Papua, Cyclops Mts., 710 m, 02°32.031'S, 140°30.412'E, local collector ca. 1997” (ZSM). *Paratypes*: 1 male with the same label as the holotype (NHMW). 1 male, 2 females “Indonesia: Papua, Cyclops Mts., 1120 m, 02°31.516'S, 140°30.436'E, local collector ca. 1997” (MZB, ZSM). 2 males, 1 female “Indonesia: Papua, Cyclops Mts., 615 m, 02°32.031'S, 140°30.412'E, local collector ca. 1997” (NHMW, ZSM). 2 males “Indonesia: Papua, Cyclops Mts., Doyo, 365 m, local collector ca. 1997” (ZSM).

#### Description.

*Body size and form*: Beetle small: TL-H 3.0–3.25 mm, TL 3.25–3.55 mm, MW 1.65–1.8 mm (holotype: TL-H 3.1 mm, TL 3.4 mm, MW 1.7 mm), with oblong-oval habitus.

*Coloration*: Reddish. Dorsal surface almost uniformly yellowish red to reddish brown, with paler anterior part of head and pronotum laterally; head appendages and legs yellowish red (Fig. 12). All type specimens are teneral, therefore, coloration may be darker.

*Surface sculpture*: Submatt dorsally. As in *E.pseudopusilla* sp. n. but microreticulation more weakly impressed, dorsal surface shinier.

*Structures*: Pronotum with lateral bead. Its lateral sides with distinct longitudinal impressions. Base of prosternum and neck of prosternal process with distinct ridge, rounded anteriorly. Blade of prosternal process lanceolate, relatively broad, slightly convex, with distinct lateral bead and few setae. Abdominal ventrite 6 slightly truncate.

*Male*: Antennae simple (Fig. 12). Protarsomere 4 with medium-sized, thick, curved anterolateral hook-like seta. Protarsomere 5 long and narrow, with anterior band of 30 and posterior row of 7 relatively long setae (Fig. 36D). Median lobe in lateral view slightly curved, with slightly thickened, straight apex; in ventral view, subparallel, very slightly narrowed distally, with broad, truncate apex. Paramere slightly concave on dorsal side, with long, dense subdistal setae, median and proximal ones finer and sparser (Fig. 36A–C). Abdominal ventrite 6 with 4–6 lateral striae on each side.

*Female*: Without evident differences in external morphology from males, except for not modified protarsi and abdominal ventrite 6 without striae.

#### Affinities.

*Exocelinacyclops* sp. n. is similar to *E.sumokedi* sp. n., *E.adelbertensis* sp. n., and *E.bewani* sp. n., but has coarser dorsal punctation. In this character, the species is more similar to *E.pusilla* sp. n. and *E.pseudopusilla* sp. n. but it differs from them in much broader and differently shaped median lobe (not distinctly narrowed distally, with apex thicker in lateral and ventral views) and from the latter, also in smaller size and slightly shinier dorsal surface.

#### Distribution.

Papua: Jayapura Regency. The species is known only from the Cyclops Mountains (Fig. 50).

#### Etymology.

The species is named after the Cyclops Mountains. The name is a noun in the nominative singular standing in apposition.

### 6. Exocelina desii

Taxon classificationAnimaliaColeopteraDytiscidae

(Balke, 1999)

Figs 15 , 39

Copelatus (Papuadytes) desii Balke, 1999: 274; Nilsson 2001: 76 (catalogue).
Papuadytes desii (Balke, 1999): Nilsson and Fery 2006: 56 (comb. n.).
Exocelina desii (Balke, 1999): Nilsson 2007: 33 (comb. n.); Nilsson and Hájek 2018: 65 (catalogue).
Exocelina desii MB1399: Toussaint et al. 2014: supplementary figs 1–4, table 2; Toussaint et al. 2015: supplementary figs S1–S2, table S3, and information S5–S6.

#### Type locality.

Papua New Guinea: East Sepik Province, Amboin Patrol Post, Karawari Lodge, ca 04°29'05.8"S, 143°26'37.5"E, < 100 m a.s.l.

#### Type material studied.

*Paratype*: female “Papau [sic!] New Guinea East Sepik Province, Amboin Patrol Post, Karawari Lodge 14Jan.1983, A.C.Messer”, “Paratypus Copelatusdesii M. Balke des. 1999” [red] (NHMW). Note: The holotype has not been found. According to Balke (1999), it was deposited in the Natural Museum of Natural History, Smithsonian Institution, Washington, DC, USA.

#### Additional material.

**Western Highlands**: 3 females “Papua New Guinea: Western Highlands, Mt. Hagen town area, 1600m, 7.xii.1994 05.49.745S 144.22.357E Balke & Kinibel (PNG 131)” (ZSM). 1 male, 1 female “Papua New Guinea: Western Highlands, Kurumul, 6Km SW Kudjip, small stream, 1584m, 13.vi.1994, 05.53.426S 144.36.600E, John (PNG 78)”, the female with an additional green label “M.Balke 1342” (ZSM). **Eastern Highlands**: 5 males “Papua New Guinea: Eastern Highlands, Akameku-Brahmin, Bismarck Range, 1900m, 23.xi.1994, 05.54.284S 145.22.271E, Balke & Kinibel (PNG 108)”, one of them with an additional green label “M.Balke 1399” (NHMW, ZSM). 3 males, 2 females “Papua New Guinea: Eastern Highlands, Akameku-Brahmin, Bismarck Range, 1500m, 24.xi.1994, 05.51.964S 145.23.604E, Balke & Kinibel (PNG 111)” (NHMW, ZSM). **Simbu**: 3 males, 1 female “Collection Naturhistorisches Museum Basel”, “Papua New Guinea Simbu prov. L. Ciek lgt.”, Kundiawa, Mu vill. 145°02'E 4°42'S III.2001, 1900m” (NHMB). 1 male “Ibisca Niugini, PNG 28–30.x.2012 Mount Wilhelm 1700m”, “-5,759269238 145,235611 FIT-MW1700-K-2/8-d01 / Plot 11 / P1959 Vial 05833” (IECA). 1 male “-5,759269238 145,235611 FIT-MW1700-J-1/8-d01 / Plot 10 / P1950 Vial 02485”, “Ibisca Niugini, PNG 25–27.x.2012 Mount Wilhelm 1700m” (ZSM). 2 females “Ibisca Niugini, PNG 27–29.x.2012 Mount Wilhelm 1700m -5,759269238 145,235611 MW1700 / P1943 Vial 04017” (ZSM). 1 female “Ibisca Niugini, PNG 31.x-2.xi.2012 Mount Wilhelm 1700m -5,759269238 145,235611 MW1700 / P1953 Vial 07522” (ZSM). 1 female “Ibisca Niugini, PNG 2–4.xi.2012 Mount Wilhelm 1700m -5,759269238 145,235611 MW1700 / P1914 Vial 07577” (ZSM). 1 female “Ibisca Niugini, PNG 25–27.x.2012 Mount Wilhelm 1700m -5,759269238 145,235611 MW1700 / P1878 Vial 02411” (ZSM). 1 female “Ibisca Niugini, PNG 5–7.xi.2012 Mount Wilhelm 1700m”, “-5,759269238 145,235611 FIT-MW1700-O-6/8-d12 / Plot 15 / P1995 Vial 05666” (ZSM). 1 female “Ibisca Niugini, PNG 27–29.x.2012 Mount Wilhelm 1700m -5,759269238 145,235611”, “FIT-MW1700-H-2/8-d03 / Plot 8 / P1935 Vial P1935-CODYTI” (ZSM). 1 female “Ibisca Niugini, PNG 27–29.x.2012 Mount Wilhelm 1700m -5,759269238 145,235611”, “FIT-MW1700-I-2/8-d03 / Plot 9 / P1943 Vial P1943-CODYTI” (ZSM). 1 female “Ibisca Niugini, PNG 28–30.x.2012 Mount Wilhelm 1700m”, “-5,759269238 145,235611 FIT-MW1700-T-2/8-d04 / Plot 20 / P2031 Vial 05553” (ZSM). 1 female “Ibisca Niugini, PNG 27–29.x.2012 Mount Wilhelm 1700m”, “-5,760916233 145,2353363 MW1700 / P1895 Vial P1895-CODYTI” (ZSM). 1 female “Ibisca Niugini, PNG 4–6.xi.2012 Mount Wilhelm 1700m”, “-5,760916233 145,2353363 FIT-MW1700-C-6/8-d11 / Plot 3 / P1899 Vial 04070” (ZSM). 1 female “Ibisca Niugini, PNG 25–27.x.2012 Mount Wilhelm 1700m -5,759910107 145,234726 MW1700 / P1886 Vial 07540” (ZSM). 1 female “Ibisca Niugini, PNG 28–30.x.2012 Mount Wilhelm 2200m”, “-5,75897789 145,1860657 FIT-MW2200-F-7/8-d13 / Plot 6 / P2314 Vial 07373-CODYTI” (ZSM). 1 female “Ibisca Niugini, PNG 27–29.x.2012 Mount Wilhelm 2200m”, “-5,75897789 145,1860657 FIT-MW2200-K-6/8-d12 / Plot 11 / P2353 Vial 11687” (ZSM). 1 female “Ibisca Niugini, PNG 28–30.x.2012 Mount Wilhelm 2200m -5,760178089 145,186264 MW2200 / P2282 Vial 07152” (ZSM). 2 females “Ibisca Niugini, PNG 29–31.x.2012 Mount Wilhelm 2200m -5,760178089 145,186264 FIT-MW2200-N-7/8-d14/ Plot 14 / P2378 Vial 15681” (ZSM). 2 females “Ibisca Niugini, PNG 27–29.x.2012 Mount Wilhelm 2200m FIT-MW2200-O-6/8-d12 / Plot 15 / P2385 Vial …-CODYTI” (ZSM). 1 female “04103 Ibisca Niugini 2012” (ZSM). 1 female “04103” (ZSM).

#### Diagnosis.

For complete description, see Balke (1999: 274). Beetle medium-sized: TL-H 3.5–4.3 mm, oblong-oval; reddish brown to brown, with paler pronotum; submatt, with dense, rather coarse punctation and rather strongly impressed microreticulation; pronotum with distinct lateral bead; male antennae simple (Fig. 15); male protarsomere 4 with anterior angle slightly expanded, with large, thick, strongly curved anterolateral hook-like seta; male protarsomere 5 slightly concave ventrally, with anterior band of ca 100 and posterior band ca 40 of relatively long setae (Fig. 39D); median lobe in lateral view evenly broad, with rounded, not curved downwards apex, in ventral view, evenly tapering, with broadly pointed apex; paramere slightly concave on dorsal side and with long, dense, thin setae, situated along dorsal margin; subdistal setae strong and dense, setae in middle part slightly shorter and sparser, proximal setae long but sparser than subdistal ones (Fig. 39A–C).

#### Affinities.

In the area of its distribution, *E.desii* co-occurs with numerous species of the *ekari*-, *ullrichi*-, *broschii*-, *larsoni*-, and *danae*-groups. From them, the species differs in size, coloration, surface sculpture, simple male antennae, and the shape of the median lobe.

Within the *casuarina*-group, it is very similar in coloration and surface sculpture to the co-occurring *E.pusilla* sp. n. and *E.pseudopusilla* sp. n. but can be distinguished from them by larger size and evidently thicker median lobe.

#### Distribution.

Papua New Guinea: East Sepik, Simbu, Western Highlands, and Eastern Highlands Provinces (Fig. 50).

### 7. Exocelina fume

Taxon classificationAnimaliaColeopteraDytiscidae

(Balke, 1998)

Figs 3 , 27

Copelatus (Papuadytes) fume Balke, 1998: 330; Nilsson 2001: 76 (catalogue).
Papuadytes fume (Balke, 1998): Nilsson and Fery 2006: 56 (comb. n.).
Exocelina fume (Balke, 1998): Nilsson 2007: 33 (comb. n.); Nilsson and Hájek 2018: 66 (catalogue).

#### Type locality.

Papua: Pegunungan Bintang Regency, Borme, 04°24'S, 140°25'E, 1800 m a.s.l.

#### Type material studied.

*Holotype*: male “IRIAN JAYA Zentralmassive 140°25'E 04°24'S, “Borme, 1800m 16.8.1992 leg. Balke (12, 12A)”, “HOLOTYPUS” [red], “Copelatusfume Balke des. 1997” [red] (NHMW). *Paratypes*: 9 males with the same label as the holotype and additionally with red labels “Paratypus Copelatusfume Balke des. 1997”, two of them with labels “M.Balke 3275” [green], and “M.Balke 3276” [green] and “M.Balke 6405” [green text] (NHMW). Note: There are two additional paratypes of *E.fume*, which do not belong to this species but to *E.ketembang* (Balke, 1998) and *E.erteldi* (Balke, 1998).

#### Diagnosis.

For complete description, see Balke (1998: 330). Beetle medium-sized: TL-H 3.7–4.4 mm; oblong-oval; brown to dark brown, with reddish brown pronotal sides, head, and sometimes also sides of elytra, in some specimens, disc of pronotum and elytron almost piceous; submatt, with fine but rather dense punctation and strongly impressed microreticulation; pronotum without lateral bead; male antennae simple (Fig. 3); male protarsomere 4 with large, thick, strongly curved anterolateral hook-like seta; male protarsomere 5 long and narrow, with anterior band of more than 60 and posterior row of 6 relatively long, thin setae (Fig. 27D); median lobe in lateral view slightly curved, with apex curved downwards, with visible angle on dorsal side, in ventral view, distally distinctly narrowed to truncate apex; paramere slightly concave on dorsal side and with long, dense, thin setae, situated along dorsal margin: subdistal setae strong and dense, setae in middle part shorter and sparser, proximal setae long, only slightly sparser than subdistal ones (Fig. 27A–C).

#### Affinities.

In the area of its distribution, *E.fume* co-occurs with *E.takime* (Balke, 1998) and species of the *ekari*-, *aipo*-, *okbapensis*-, *aipomek*-, *erteldi*-, and *danae*-groups. From species of the *ekari*-group, the species differs in larger size, evidently stronger dorsal punctation and microreticulation, and the shape of the median lobe. In the latter two characters, *E.fume* differs also from the species of the remaining groups, as well as in absence of the pronotal bead and simple male antennae.

Within the *casuarina*-group, the species is more similar to *E.casuarina* and *E.ibalimi* sp. n., especially the latter one, from which can be distinguished by paler coloration and the shape of the median lobe (see their “Affinities” and the “Key”).

#### Distribution.

Papua: Pegunungan Bintang. The species is known only from the type locality (Fig. 50).

### 8. Exocelina heidiae

Taxon classificationAnimaliaColeopteraDytiscidae

(Balke, 1998)

Figs 16 , 40

Copelatus (Papuadytes) heidiae Balke, 1998: 331; Nilsson 2001: 76 (catalogue).
Papuadytes heidiae (Balke, 1998): Nilsson and Fery 2006: 56 (comb.n.).
Exocelina heidiae (Balke, 1998): Nilsson 2007: 33 (comb. n.); Nilsson and Hájek 2018: 66 (catalogue).

#### Type locality.

Papua New Guinea: Morobe Province, Herzog Range, Wagau (Vagau), ca 06°48'S, 146°48'E, ca 1300 m a.s.l.

#### Type material studied.

*Paratypes*: 3 males “Stn. No. 150”, “New Guinea: Morobe Dist., Herzog Mts., Vagau, C.4,000ft. 4–17.i.1965”, “M. E. Bacchus. B. M. 1965–120”, “Paratypus Copelatusheidiae sp.n. Balke des. 1997” [red] (NHMW). 1 female “Stn. No. 140A”, “New Guinea: Morobe Dist., Herzog Mts., Vagau, C.4,000ft. 4–17.i.1965”, “M. E. Bacchus. B. M. 1965–120”, “Paratypus Copelatusheidiae sp.n. Balke des. 1997” [red] (NHMW).

#### Diagnosis.

For complete description, see Balke (1998: 331). Beetle medium-sized: TL-H 4.35–4.9 mm; broader, oblong-oval; dark brown, with reddish brown pronotal sides and head anteriorly, in some specimens, disc of pronotum and elytron almost piceous; submatt, with very fine, on elytra often almost invisible punctation and strongly impressed microreticulation; pronotum with lateral bead; male antennae simple (Fig. 16); male protarsomere 4 with anterior angle very slightly expanded, with large, thick, strongly curved anterolateral hook-like seta; male protarsomere 5 long and narrow, slightly concave ventrally, with anterior band of ca 70 and posterior band of ca 30 relatively long setae (Fig. 40D); median lobe in lateral view evenly broad, with rounded, not curved downwards, only slightly thickened apex, in ventral view, with subparallel sides and roundly truncate apex; paramere slightly concave on dorsal side and with long, dense, thin setae, situated along dorsal margin distinctly divided to dense and strong subdistal setae and sparser proximal ones, setae in middle short and fine (Fig. 40A–C).

#### Affinities.

In the Herzog Range area, *E.heidiae* co-occurs with Exocelina*jasminae* (Balke, 1998), two species of the *ekari*-group, and four species of the *danae*-group. From all of them, the species differs in larger size and the shape of the median lobe. Additional characters for the species separations are presence of the pronotal bead, simple male antennae, and dorsal punctation and microreticulation.

Within the *casuarina*-group, the species is more similar to *E.simbaijimi* sp. n. (see its “Affinities” and the “Key”).

#### Distribution.

Papua New Guinea: Morobe Province. The species is known only from the type locality, Wagau in Herzog Range (Fig. 50).

### 9. Exocelina ibalimi

Taxon classificationAnimaliaColeopteraDytiscidae

Shaverdo & Balke sp. n.

http://zoobank.org/F2B4E65B-545D-4F76-8ED4-6D9C43DA6CAB

Figs 4 , 28

Exocelina undescribed sp. MB0657: Toussaint et al. 2014: supplementary figs 1–4, table 2; Toussaint et al. 2015: supplementary figs S1–S2, table S3.
Exocelina ibalimi _New_Guinea_MB0657: Toussaint et al. 2015: Supplementary information S5–S6.

#### Type locality.

Papua New Guinea: Sandaun Province, Mianmin area, ca 04°55.78'S, 141°38.18'E, 1080 m a.s.l.

#### Type material.

*Holotype*: male “Papua New Guinea: Sandaun, Mianmin area, >600m, 13.i.2010, Ibalim & Pius (PNG235)” (ZSM). *Paratypes*: 15 males, 14 females with the same label as the holotype, 2 males with additional labels “M.Balke 4929”, “M.Balke 4932”, and one female with “M.Balke 4930” (NHMW, ZSM). 4 males, 1 female “Papua New Guinea: Sandaun, Ofektaman, 820m, 17.x.2008, 5.04.113S 141.35.841E, Ibalim (PNG 190)”, two males additionally with green labels “M.Balke 3724”, “M.Balke 3725” (NHMW, ZSM). 3 females “Papua New Guinea: Sandaun, Mianmin, 670m 20.x.2008, 4.53.292S 141.34.118E, Ibalim (PNG 191)” (ZSM). 2 males “Papua New Guinea: Sandaun, Mianmin (river), 990m, 23.x.2008, 4.54.570S 141.35.490E, Ibalim (PNG 192)” (ZSM). 3 males “Papua New Guinea: Sandaun, Mianmin (pool), 990m, 23.x.2008, 4.54.570S 141.35.490E, Ibalim (PNG 193)”, two of them with additional green labels “M.Balke 3739”, “M.Balke 3740” (ZSM). 4 males, 4 females “Papua New Guinea: Sandaun, Mianmin (river), 1080m, 24.x.2008, 04.55.780S 141.38.185E, Ibalim (PNG 195)” (NHMW, ZSM). 6 males, 12 females “Papua New Guinea: Sandaun, Mianmin (pool), 1080m, 24.x.2008, 04.55.780S 141.38.185E, Ibalim (PNG 196)” (ZSM). 2 males, 1 female “Papua New Guinea: Sandaun, Mianmin (river) 700m, 21.x.2008, 04.52.858S 141.31.706E Ibalim (PNG 197)” (ZSM). 5 males, 3 females “Papua New Guinea: Sandaun, Mianmin (pool), 700m, 21.x.2008, 04.52.858S 141.31.706E, Ibalim (PNG 198)” (NHMW, ZSM). 2 males “Papua New Guinea: Sandaun, Mianmin area, >1000m, 23.xii.2009, Ibalim & Pius (PNG232)” (ZSM). 4 males, 1 female “Papua New Guinea: Sandaun, Mianmin area, >1000m, 26.xii.2009, Ibalim & Pius (PNG233)” (NHMW, ZSM). 8 males, 3 females “Papua New Guinea: Sandaun, Mianmin area, >600m, 13.i.2010, Ibalim & Pius (PNG236)”, one of them with “M.Balke 4925” (NHMW, ZSM). 5 males, 8 females “Papua New Guinea: Sandaun, Mianmin area, >700m, 14.i.2010, 0 4 54.540S 141 36.953E, Ibalim & Pius (PNG238)” (NHMW, ZSM). 1 male “Papua New Guinea: Sandaun, Mianmin 2, 1150 m, 20.x.2003, 04 52.562S 141 37.038E, K. Sagata (WB70)”, “M. Balke 658” [green text] (ZSM). 2 males “Papua New Guinea: Sandaun, MekilK [!], 1718m, 14.x.2003, 4 48.742S 141 39.075E, K. Sagata (WB106)”, one of them with “M. Balke 661” [green text] (ZSM). 1 male “Papua New Guinea: Sandaun: Mekil (WB106), 14.x.2003, K. Sagata, M Balke: MB 660”, “M. Balke 660” [green text] (ZSM). 1 female “Papua New Guinea: Sandaun, Sokamin village, 1200m, 9.x.2003, 4 51.883S 141 37.534E, K. Sagata (WB97)”, “M. Balke 657” [green text] (ZSM). 2 males, 1 female “Papua New Guinea: Sandaun, Sokamin4, 1200m, 19.x.2003, 4 50.845S 141 37.865E, K. Sagata (WB100)”, males with green text labels “M. Balke 683” and “M. Balke 684” (ZSM).

#### Description.

*Body size and form*: Beetle size variable but generally beetle medium-sized: TL-H 3.45–4.3 mm, TL 3.75–4.8 mm, MW 1.8–2.25 mm (holotype: TL-H 4.15 mm, TL 3.75 mm, MW 2 mm), with oblong-oval habitus, slightly more attenuated posteriorly.

*Coloration*: Brown to piceous, with head and pronotum paler. Head reddish brown to piceous, darker posteriorly. pronotum reddish brown to piceous, broadly paler on lateral sides and sometimes also narrowly anteriorly and posteriorly. Elytra uniformly brown to piceous. Head appendages and legs yellowish red to reddish brown, legs distally darker, especially metathoracic legs (Fig. 4). Teneral specimens paler.

*Surface sculpture*: Submatt dorsally. Head with rather dense punctation (spaces between punctures 1–2 times size of punctures), evidently finer and sparser anteriorly; diameter of punctures smaller than diameter of cells of microreticulation. Pronotum and elytra with fine but rather dense punctation, sparser and finer than on head. Pronotum and elytra with strongly impressed microreticulation. Head with microreticulation stronger. Metaventrite and metacoxae distinctly microreticulate, metacoxal plates with longitudinal strioles and transverse wrinkles. Abdominal ventrites with distinct microreticulation, strioles, and very fine sparse punctation.

*Structures*: Pronotum without lateral bead. Base of prosternum and neck of prosternal process with distinct ridge, rounded anteriorly. Blade of prosternal process lanceolate, relatively narrow, slightly convex, with distinct lateral bead and few setae. Abdominal ventrite 6 broadly rounded or slightly truncate.

*Male*: Antennae simple (Fig. 4). Protarsomere 4 with large, thick, strongly curved anterolateral hook-like seta. Protarsomere 5 long and narrow, with anterior band of more than 80 and posterior row of 11 relatively long, thin setae (Fig. 28D). Median lobe in lateral view slightly curved, its apex rounded and not or only very slightly curved downwards; in ventral view, distally distinctly narrowed to truncate apex. Paramere slightly concave on dorsal side and with long, dense, thin setae situated along dorsal margin: subdistal setae denser, proximal setae sparser, setae in middle shorter, thinner (Fig. 28A–C). Abdominal ventrite 6 with 7–11 lateral striae on each side.

*Female*: Without evident differences in external morphology from males, except for not modified protarsi and abdominal ventrite 6 without striae.

#### Variability.

The species has variability in size, coloration and shape of the median lobe. Beetles are small to medium-sized (see the measurements above) and with coloration: from reddish head and pronotum and dark brown elytra to uniformly piceous with reddish brown pronotal sides. Median lobe shows different shape of its apex: in lateral view, it is not curved or differently slightly curved downwards reminding that of *E.fume* but without distinct angle.

#### Affinities.

*Exocelinaibalimi* sp. n. is very similar to *E.fume* but differs from it in shape of the median lobe: its apex not curved or only slightly curved downwards, more or less rounded in lateral view, without distinct angle on the dorsal side. The species also has dorsal punctation distinctly finer and microreticulation less strongly impressed than in *E.fume*.

#### Distribution.

Papua New Guinea: Sandaun Province (Fig. 50).

#### Etymology.

The species is named for Sentiko Ibalim, one of the great young PNG entomologists, who collected most of these beetles. The species name is a noun in the genitive case.

### 10. Exocelina keki

Taxon classificationAnimaliaColeopteraDytiscidae

Shaverdo & Balke sp. n.

http://zoobank.org/0C2652F3-452D-4826-918A-90D6C5C5ECA8

Figs 6 , 30

Exocelina undescribed sp. MB1530: Toussaint et al. 2014: supplementary figs 1–4, table 2; Toussaint et al. 2015: supplementary figs S1–S2, table S3.
Exocelina pseudokeki _New_Guinea_MB1530: Toussaint et al. 2015: supplementary information S5–S6.

#### Type locality.

Papua New Guinea: Madang, Adelbert Mts, creek near Keki, 04°42.30'S, 145°25.09'E, 790 m a.s.l.

#### Type material.

*Holotype*: male “Papua New Guinea: Madang, Keki, Adalbert [sic!] Mts., 500m, 29.xi.2006, nr 04.43.058S 145.24.437E, Balke & Kinibel (PNG 118)” (ZSM). *Paratypes*: **Madang**: 9 males, 4 females with the same labels as the holotype, one male with an additional green label “M.Balke 1530” (NHMW, ZSM). 1 male, 3 females “Papua New Guinea: Madang, Adalbert [sic!] Mts., creek nr Keki, 790m, 28.xi.1994, 04.42.300S 145.25.089E, Binatang Boys leg. (PNG 53a)” (ZSM). 1 male, 2 females “Papua New Guinea: Madang, Keki-Sewan, Adalbert [sic!] Mts., 700m, 30.xi.2006, nr 04.41.802S 145.25.460E, Binatang Boys (PNG 120)” (ZSM). **Eastern Highlands**: 1 male “Papua New Guinea: Eastern Highlands, Bena Bridge, 1400m, 8.xii.2007, 06.10.781S 145.26.034E, Balke & Sagata (PNG 164)” (ZSM).

#### Description.

*Body size and form*: Beetle small: TL-H 3.15–3.65 mm, TL 3.45–4.0 mm, MW 1.65–1.9 mm (holotype: TL-H 3.65 mm, TL 4 mm, MW 1.85 mm), with oblong habitus.

*Coloration*: Reddish to reddish brown, with head and pronotum paler. Head yellowish red to reddish brown, with small darker areas posterior to eyes. Pronotum yellowish red to reddish brown, with small brown to dark brown area on disc. Elytra reddish brown to brown, with narrow reddish sutural lines. Head appendages yellowish red, legs reddish, distally darker, especially metathoracic legs (Fig. 6). Teneral specimens paler.

*Surface sculpture*: Submatt dorsally. Head with rather dense punctation (spaces between punctures 1–2 times size of punctures), evidently finer and sparser anteriorly; diameter of punctures smaller than diameter of cells of microreticulation. Pronotum and elytra with dense, distinct but fine punctation, sparser and finer than on head. Pronotum and elytra with strongly impressed microreticulation. Head with microreticulation stronger. Metaventrite and metacoxae distinctly microreticulate, metacoxal plates with longitudinal strioles and transverse wrinkles. Abdominal ventrites with distinct microreticulation, strioles, and very fine sparse punctation.

*Structures*: Pronotum without lateral bead, in few specimens with its traces in posterior part. Base of prosternum and neck of prosternal process with distinct ridge, slightly rounded anteriorly. Blade of prosternal process lanceolate, relatively narrow, slightly convex, with distinct lateral bead and few setae. Abdominal ventrite 6 rounded.

*Male*: Antennae simple (Fig. 6). Protarsomere 4 with anterolateral seta thin, long, smaller than more laterally situated large setae, slightly curved downwards. Protarsomere 5 long and narrow, with anterior band of more than 40 and posterior row of 7 relatively long, thin setae (Fig. 30D). Median lobe in lateral view almost straight, its apex rounded and not curved downwards; in ventral view, distally distinctly narrowed before rounded, narrow apex. Paramere slightly concave on dorsal side and with dorsal setae distinctly divided to long, dense subdistal setae and sparser proximal ones (Fig. 30A–C). Abdominal ventrite 6 with 3–5 lateral striae on each side.

*Female*: Without evident differences in external morphology from males, except for not modified protarsi and abdominal ventrite 6 without striae.

#### Distribution and variability.

Papua New Guinea: Madang and Eastern Highlands (Fig. 50). The species is known mainly from Keki area in Adelbert Mountains; only one beetle was collected in Bena, Eastern Highlands. It shows no morphological difference from the specimens of Keki populations, except for a small difference in the median lobe shape, which could be an expression of species variability. The species might have the same pattern of distribution as *Exocelinabrahminensis*Shaverdo et al., 2012, which has a wide distribution in the Momase Region and is known from Adelbert Mountains and Bena.

#### Affinities.

*Exocelinakeki* sp. n. is very similar to *Exocelinamesseri* (Balke, 1999) in body form and coloration, but has much more distinct dorsal punctation and stronger microreticulation, as well as median lobe more slender, with apex smaller and narrower in ventral view; the ventral setae of male protarsomere 5 are much less numerous and clearly divided into anterior band and posterior row.

#### Etymology. The

species is named after Keki Village. The name is a noun in the nominative singular standing in apposition.

### 11. Exocelina kumulensis

Taxon classificationAnimaliaColeopteraDytiscidae

Shaverdo & Balke sp. n.

http://zoobank.org/B665E92E-4CB0-4D47-87DF-08DAFAFFA4F2

Figs 25 , 49

Exocelina undescribed sp. MB1360: Toussaint et al. 2014: supplementary figs 1–4, table 2; Toussaint et al. 2015: supplementary figs S1–S2, table S3.
Exocelina hagenensis _New_Guinea_MB1360: Toussaint et al. 2015: supplementary information S5–S6.

#### Type locality.

Papua New Guinea: Enga Province, Kumul Lodge at foot of Mt Hagen, 05°47.55'S, 143°58.76'E, 2700 m a.s.l.

#### Type material.

*Holotype*: male “M. Balke 1360” [green], “Papua New Guinea: Enga, Kumul Lodge @ foot of Mt. Hagen, 2700m, 5.xii.2006, 05.47.548S 143.58.761E, Balke & Kinibel (PNG 124)” (ZSM).

#### Description.

*Body size and form*: Beetle large: TL-H 5.4 mm, TL 6.0 mm, MW 2.9 mm, with broader, oblong-oval habitus.

*Coloration*: Piceous, with paler pronotum. Head piceous, narrowly brownish anteriorly and with two vague brownish spots between eyes. Pronotum dark brown, piceous on disc. Elytra piceous, with vague narrow brownish sutural lines. Head appendages and legs proximally reddish brown, legs distally darker, brownish, especially metathoracic legs (Fig. 25).

*Surface sculpture*: Submatt dorsally. Head with dense, coarse punctation (no spaces between punctures or spaces of equal size of punctures), finer and sparser anteriorly; diameter of punctures equal to diameter of cells of microreticulation. Pronotum with relatively dense but fine punctation, sparser and finer than on head. Elytra with finer punctation than on pronotum. Pronotum and elytra with strongly impressed microreticulation. Head with microreticulation stronger. Metaventrite and metacoxae distinctly microreticulate, metacoxal plates with longitudinal strioles and transverse wrinkles, abdominal ventrites with distinct microreticulation and strioles. Metaventrite medially, metacoxal plates, and abdominal ventrites with fine, sparse punctation.

*Structures*: Pronotum with distinct lateral bead. Its lateral sides with distinct longitudinal impressions. Base of prosternum and neck of prosternal process with distinct ridge, slightly rounded anteriorly. Blade of prosternal process lanceolate, relatively short, broad, slightly convex and smooth in the middle, with distinct lateral bead and few lateral setae, lateral sides flattened. Abdominal ventrite 6 rounded.

*Male*: Antennae simple (Fig. 25). Protarsomere 4 with anterior angle slightly expanded, with large, thick, strongly curved anterolateral hook-like seta. Protarsomere 5 slightly concave ventrally, with anterior band of ca 100 and posterior band of ca 40 relatively long setae (Fig. 49D). Median lobe in lateral view long, slightly curved, with small, very slightly bent downwards, thickened apex; in ventral view, evenly tapering to broadly pointed apex. Paramere slightly concave on dorsal side and with weak dorsal setation, setae on subdistal part stronger, denser, more evident than proximal setae (Fig. 49A–C). Abdominal ventrite 6 with 17–18 lateral striae on each side.

*Female*: Unknown.

#### Affinities.

*Exocelinakumulensis* sp. n. is similar to *E.mendiensis* sp. n. but differs from it by being larger, having coarser and denser dorsal punctation and by the shape of the median lobe.

#### Distribution.

Papua New Guinea: Enga Province. The species is known only from the type locality (Fig. 50).

#### Etymology.

The species is named after Kumul Lodge. The name is an adjective in the nominative singular.

### 12. Exocelina mendiensis

Taxon classificationAnimaliaColeopteraDytiscidae

Shaverdo & Balke sp. n.

http://zoobank.org/5703539F-668B-4E41-A659-CCA7FD2884DA

Figs 24 , 48

Exocelina undescribed sp. MB1337: Toussaint et al. 2014: supplementary figs 1–4, table 2; Toussaint et al. 2015: supplementary figs S1–S2, table S3.
Exocelina mendiensis _New_Guinea_MB1337: Toussaint et al. 2015: supplementary information S5–S6.

#### Type locality.

Papua New Guinea: Southern Highlands Province, Sopulkul, 30–35 km NE Mendi, 06°02.94'S, 143°46.49'E, 2680 m a.s.l.

#### Type material.

*Holotype*: male “M. Balke 1337”, “Papua New Guinea: Southern Highlands, Sopulkul, 30–35 km NE Mendi, 2680 m, 16.vi.2006, 06.02.944S 143.46.485E, John (PNG 79)” (ZSM). *Paratypes*: 1 male, 2 females with the same label as the holotype (NHMW, ZSM).

#### Description.

*Body size and form*: Beetle large: TL-H 4.8–5.5 mm, TL 5.3–5.9 mm, MW 2.6–2.75 mm (holotype: TL-H 4.8 mm, TL 5.3 mm, MW 2.6 mm), with broader, oblong-oval habitus.

*Coloration*: Piceous. Head piceous, narrowly brownish anteriorly and sometimes with two brownish spots between eyes. Pronotum piceous, brownish laterally and anteriorly. Elytra piceous, sometime with narrow brownish sutural lines. Head appendages and legs proximally reddish brown, legs distally darker, brownish, especially metathoracic legs (Fig. 24).

*Surface sculpture*: Submatt dorsally. Head with relatively dense punctation (no spaces between punctures or spaces 2 times size of punctures), sparser anteriorly, denser and coarser between eyes; diameter of punctures smaller than or equal to diameter of cells of microreticulation. Pronotum with relatively dense but fine punctation, sparser and finer than on head. Elytra with very fine, sparse punctation. Pronotum and elytra with rather strongly impressed microreticulation. Head with microreticulation stronger. Metaventrite and metacoxae distinctly microreticulate, metacoxal plates with longitudinal strioles and transverse wrinkles, abdominal ventrites with distinct microreticulation and strioles. Metaventrite medially, metacoxal plates, and abdominal ventrites with very fine, sparse punctation.

*Structures*: Pronotum with distinct lateral bead. Its lateral sides with distinct longitudinal impressions. Base of prosternum and neck of prosternal process with distinct ridge, slightly rounded anteriorly. Blade of prosternal process lanceolate, relatively narrow, slightly convex, and smooth, with distinct lateral bead and few lateral setae. Abdominal ventrite 6 slightly truncate.

*Male*: Antennae simple (Fig. 24). Protarsomere 4 with anterior angle slightly expanded, with large, thick, strongly curved anterolateral hook-like seta. Protarsomere 5 with anterior band of more than 60 and posterior row of 16 relatively long setae (Fig. 48D). Median lobe in lateral view long, slightly curved, with small, slightly bent downwards, thickened apex; in ventral view, evenly tapering to broadly pointed apex. Paramere slightly concave on dorsal side and with weak dorsal setation, setae on subdistal part stronger and denser than proximal setae (Fig. 48A–C). Abdominal ventrite 6 with 23–27 lateral striae on each side.

*Female*: Without evident differences in external morphology from males, except for not modified protarsi and abdominal ventrite 6 without striae.

#### Affinities.

*Exocelinamendiensis* sp. n. is similar to the larger species of the group, *E.kumulensis* sp. n. and *E.ambua* sp. n., but differs from them in its darker coloration, shinier dorsal surface and shape of the median lobe. Also, see under *E.okapa* sp. n.

#### Distribution.

Papua New Guinea: Southern Highlands Province. The species is known only from the type locality (Fig. 50).

#### Etymology.

The species is named after Mendi Village. The name is an adjective in the nominative singular.

### 13. Exocelina menyamya

Taxon classificationAnimaliaColeopteraDytiscidae

Shaverdo & Balke sp. n.

http://zoobank.org/B6435BF0-3067-44BD-946E-8772C350CAD1

Figs 20 , 44

Exocelina undescribed sp. MB1377: Toussaint et al. 2014: supplementary figs 1–4, table 2; Toussaint et al. 2015: supplementary figs S1–S2, table S3.
Exocelina menyamya _New_Guinea_MB1377: Toussaint et al. 2015: supplementary information S5–S6.

#### Type locality.

Papua New Guinea: Morobe Province, Menyamya, Mount Inji, 07°14.26'S, 146°01.40'E, 1500 m a.s.l.

#### Type material.

*Holotype*: male “Papua New Guinea: Morobe, Menyamya, Mt Inji, deep well, 1500m, 14.xi.2006, 07.14.264S 146.01.400E, Balke & Kinibel (PNG 98)”, “M.Balke 1377” [green] (ZSM).

#### Description.

*Body size and form*: Beetle medium-sized: TL-H 4.25 mm, TL 4.8 mm, MW 2.2 mm, with oblong-oval habitus.

*Coloration*: Brown, with reddish pronotum. Head brown, with slightly darker areas posterior to eyes. Pronotum broadly reddish laterally and dark brown medially from anterior to posterior margins. Elytra uniformly brown, in the middle with traces of narrow reddish sutural lines. Head appendages and legs proximally yellowish red, legs distally darker, reddish brown, especially metathoracic legs (Fig. 20).

*Surface sculpture*: Matt dorsally. Head with rather dense, coarse punctation (spaces between punctures 1–2 times size of punctures), evidently finer and sparser anteriorly; diameter of punctures smaller than or equal to diameter of cells of microreticulation. Pronotum and elytra with distinct punctation, sparser and finer than on head. Pronotum and elytra with strongly impressed microreticulation. Head with microreticulation stronger. Metaventrite and metacoxae distinctly microreticulate, metacoxal plates with longitudinal strioles and transverse wrinkles. Abdominal ventrites with distinct microreticulation, strioles, and very fine sparse punctation.

*Structures*: Pronotum with distinct lateral bead. Its lateral sides with longitudinal impressions. Base of prosternum and neck of prosternal process with distinct ridge, slightly rounded anteriorly. Blade of prosternal process lanceolate, relatively short, broad, slightly convex and smooth in the middle, with distinct lateral bead and few lateral setae, lateral sides slightly flattened. Abdominal ventrite 6 slightly truncate.

*Male*: Antennae simple (Fig. 20). Protarsomere 4 with large, thick, slightly curved anterolateral hook-like seta. Protarsomere 5 ventrally with anterior band of more than 50 and posterior row of 13 relatively long, thin setae (Fig. 44D). Median lobe in lateral view narrowed to apex, with thin, slightly curved upwards apex; in ventral view, narrowed before apex, with apex rounded, of shape of a baby pacifier, with distinct ventral impression. Paramere slightly concave on dorsal side and with long, dense, thin setae situated along dorsal margin, subdistal setae denser, proximal setae much sparser, setae in middle shorter, thinner (Fig. 44A–C). Abdominal ventrite 6 with 19–22 lateral striae on each side.

*Female*: Unknown.

#### Affinities.

*Exocelinamenyamya* sp. n. is similar to *E.casuarina* and *E.fume* in body size, shape, and coloration but can be distinguished from them by the completely different shape of the median lobe and presence of the pronotal bead.

#### Distribution.

Papua New Guinea: Morobe Province. The species is known only from the type locality (Fig. 50).

#### Etymology.

The species is named after Menyamya Village. The name is a noun in the nominative singular standing in apposition.

### 14. Exocelina messeri

Taxon classificationAnimaliaColeopteraDytiscidae

(Balke, 1999)

Figs 5 , 29

Copelatus (Papuadytes) messeri Balke, 1999: 274; Nilsson 2001: 77 (catalogue).
Papuadytes messeri (Balke, 1999): Nilsson and Fery 2006: 56 (comb. n.).
Exocelina messeri (Balke 1999): Nilsson 2007: 34 (comb. n.); Nilsson and Hájek 2018: 67 (catalogue).

#### Type locality.

Papua New Guinea: East Sepik Province, Amboin Patrol Post, Karawari Lodge.

#### Type material studied.

*Paratypes*: 2 males “Papua New Guinea: East Sepik Province, Amboin Patrol Post, Karawari Lodge, 7 Feb.1983, A.C. Messer”, “Paratypus *Copelatusmesseri* Balke des. 1999” [red] (NHMW). Note: The holotype has not been found. According to Balke (1999), it was deposited in the Natural Museum of Natural History, Smithsonian Institution, Washington, DC, USA.

#### Diagnosis.

**Figures 5–8.**
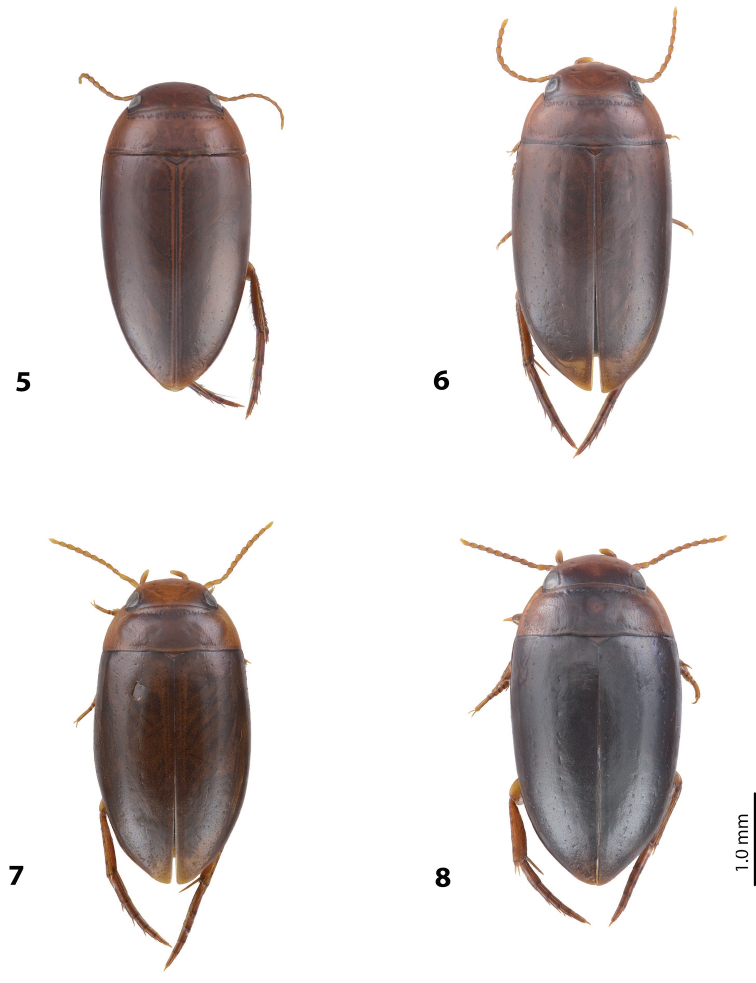
Habitus and coloration **5***Exocelinamesseri* (Balke, 1999) **6***E.keki* sp. n. **7***E.pseudofume* sp. n. **8***E.sima* sp. n.

For complete description, see Balke (1999: 274–275). Beetle small: TL-H 3.2–3.7 mm; oblong-oval, more strongly attenuated posteriorly; reddish to reddish brown, with head and pronotum slightly paler; shiny, with very fine, sparse punctation, almost invisible on elytra and weakly impressed microreticulation; pronotum without lateral bead, sometimes with its traces in posterior part; male antennae simple (Fig. 5); male protarsomere 4 with anterolateral seta thin, long, smaller than more laterally situated large setae, slightly curved downwards; male protarsomere 5 long and narrow, with more than 80 relatively long, thin setae, which divided in anterior and posterior ones proximally and mixed up together in distal half of tarsomere (Fig. 29D); median lobe in lateral view almost straight, its apex rounded and not curved downwards, in ventral view, distally narrowed before apex, apex broad, slightly rounded; paramere slightly concave on dorsal side and with dorsal setae distinctly divided into long, dense subdistal setae and sparser, rather inconspicuous proximal ones, setae in middle part short and fine (Fig. 29A–C).

#### Affinities.

See under *E.keki* sp. n.

**Distribution.** Papua New Guinea: East Sepik Province. The species is known only from the type locality (Fig. 50).

### 15. Exocelina okapa

Taxon classificationAnimaliaColeopteraDytiscidae

Shaverdo & Balke sp. n.

http://zoobank.org/A1C1E56D-459F-4BF1-A670-7EDE937644FA

Figs 23 , 47

#### Type locality.

Papua New Guinea: Eastern Highlands Province, Wapi Creek, Kimiagomo, Okapa, 06°25.41'S, 145°34.48'E, 1900 m a.s.l.

#### Type material.

*Holotype*: male “Papua New Guinea: Eastern Highlands, Wapi Creek, Kimiagomo, Okapa, 1900m, 9.viii.2005, 6 25.407S 145 34.480E, K.Sagata (WB122)” (ZSM). *Paratypes*: 5 males, 2 females with the same label as the holotype (NHMW, ZSM). 1 male, 3 females “Papua New Guinea: Eastern Highlands, Tegupate creek Kimiagomo, Okapa, 1900m, 9.viii.2005, 6 25.407S 145 34.480E, K.Sagata (WB124)” (NHMW, ZSM). 1 male, 2 females “Papua New Guinea: Eastern Highlands, Kimiagomo vill, north Okapa stn, 1900, 30.iv.2006, 06.25.407S 145.34.480E, Sagata (PNG 80)” (NHMW, ZSM). 1 male, 4 females “Papua New Guinea: Eastern Highlands, Yuyulio, Kimiagomo-Okapa, 2100m, 13.iv.2003, 06 25.255S 145 34.233E, K. Sagata (WB7)” (NHMW, ZSM). 1 male “Papua New Guinea: Eastern Highlands, Kainantu, Yoginofi, 1900m, 9.v.1994, 06.21.799S 145.45.463E, Balke & Sagata (PNG 55)” (ZSM).

#### Additional material.

3 females “Papua New Guinea: Eastern Highlands, Hano kotu, Kimiagomo, Okapa, 1661m, 11.viii.2006, 06 25.096S 145 34.556E, K.Sagata (WB129)” (ZSM).

#### Description.

*Body size and form*: Beetle medium-sized: TL-H 3.95–4.7 mm, TL 4.3–5.05 mm, MW 2.05–2.5 mm (holotype: TL-H 4.3 mm, TL 4.7 mm, MW 2.3 mm), with oblong-oval habitus, slightly more attenuated posteriorly.

*Coloration*: Piceous, with reddish brown pronotum. Head reddish brown to piceous, paler anteriorly and darker posterior to eyes. Pronotum dark brown to piceous, with reddish to reddish brown sides narrowly or broadly. Elytra dark brown to piceous, sometime with narrow reddish sutural lines. Head appendages and legs proximally yellowish red, legs distally darker, reddish brown, especially metathoracic legs (Fig. 23). Teneral specimens paler.

*Surface sculpture*: Shiny dorsally. Head mostly with fine, sparse punctation (spaces between punctures 2–3 times size of punctures) but punctation denser and coarser between eyes; diameter of punctures smaller than diameter of cells of microreticulation. Pronotum and elytra with very fine, sparse punctation, sometimes inconspicuous on elytra. Pronotum and elytra with weakly impressed microreticulation. Head with microreticulation stronger. Metaventrite and metacoxae distinctly microreticulate, metacoxal plates with longitudinal strioles and transverse wrinkles, abdominal ventrites with distinct microreticulation and strioles. Metaventrite medially, metacoxal plates, and abdominal ventrites with very fine, sparse, often inconspicuous punctation.

*Structures*: Pronotum with distinct lateral bead. Its lateral sides with distinct longitudinal impressions. Base of prosternum and neck of prosternal process with distinct ridge, slightly rounded anteriorly. Blade of prosternal process lanceolate, relatively narrow, slightly convex, and smooth, with distinct lateral bead and few lateral setae. Abdominal ventrite 6 rounded.

*Male*: Antennae simple (Fig. 23). Protarsomere 4 with large, thick, strongly curved anterolateral hook-like seta. Protarsomere 5 ventrally with anterior band of ca 60 and posterior row of 20 relatively long, thin setae (Fig. 47D). Median lobe in lateral view slightly curved, with apex dully pointed, slightly bent downwards; in ventral view, broadened subdistally, with broad, rounded apex. Paramere slightly concave on dorsal side and with dense setae on subdistal part; proximal setae inconspicuous (Fig. 47A–C). Abdominal ventrite 6 with 8–12 lateral striae on each side.

*Female*: Without evident differences in external morphology from males, except for not modified protarsi and abdominal ventrite 6 without striae.

#### Affinities.

*Exocelinaokapa* sp. n. is similar to *E.yoginofi* sp. n. but differs from it in very weak dorsal punctation and microreticulation and more slender median lobe. The species is also similar to *E.mendiensis* sp. n. but differs from it in distinctly smaller size and differently shaped apex of the median lobe.

#### Distribution.

Papua New Guinea: Eastern Highlands Province (Fig. 50).

#### Etymology.

The species is named after Okapa Station. The name is a noun in the nominative singular standing in apposition.

### 16. Exocelina piusi

Taxon classificationAnimaliaColeopteraDytiscidae

Shaverdo & Balke sp. n.

http://zoobank.org/A1C1E56D-459F-4BF1-A670-7EDE937644FA

Figs 9 , 33

Exocelina undescribed sp. MB4921: Toussaint et al. 2014: supplementary figs 1–4, table 2; Toussaint et al. 2015: supplementary figs S1–S2, table S3.
Exocelina piusi _New_Guinea_MB4921: Toussaint et al. 2015: supplementary information S5–S6.

#### Type locality.

Papua New Guinea: East Sepik Province, Lembena, 04°56.859'S, 143°59.375'E, 1,250 m a.s.l.

#### Type material.

*Holotype*: male “Papua New Guinea: East Sepik, Lembena, 335m, 10.ix.2009, 04 56.859S 143 59.375E, Ibalim & Pius (PNG250)”, “M. Balke 4920” (ZSM). *Paratype*: 1 male with the same label as the holotype, “M. Balke 4921” (ZSM).

#### Description.

*Body size and form*: Beetle small: TL-H 3.5–3.6 mm, TL 3.9–4.0 mm, MW 1.95–2.0 mm (holotype: TL-H 3.6 mm, TL 4 mm, MW 1.95 mm), with oblong-oval habitus.

**Figures 9–14.**
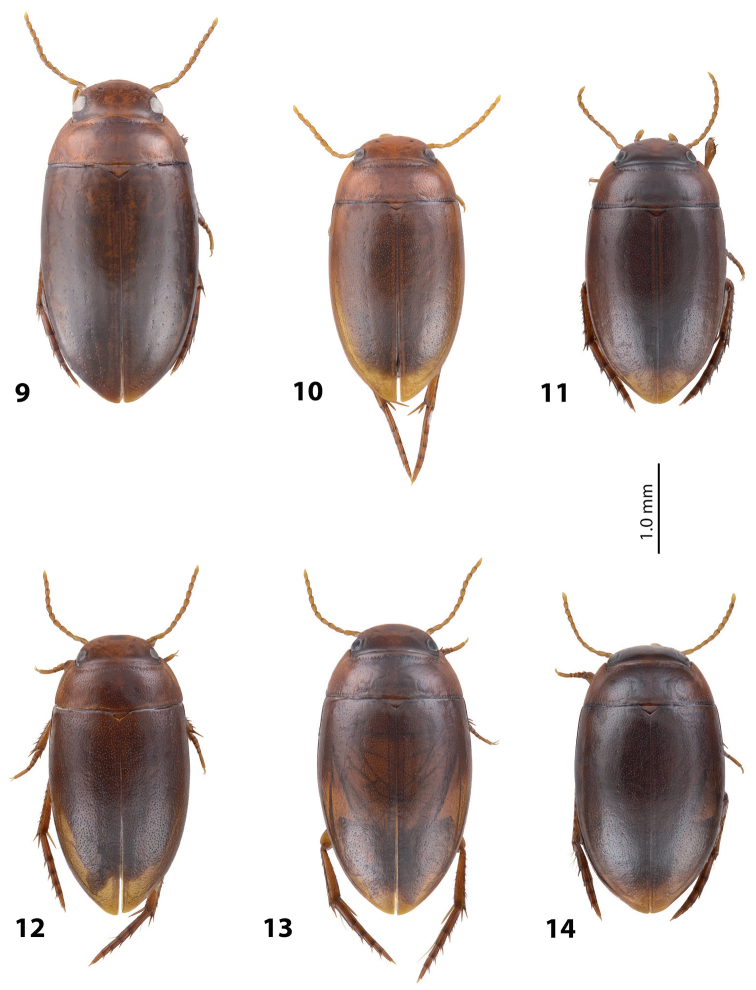
Habitus and coloration **9***Exocelinapiusi* sp. n. **10***E.pusilla* sp. n. **11***E.sumokedi* sp. n. **12***E.cyclops* sp. n. **13***E.bewani* sp. n. **14***E.adelbertensis* sp. n.

*Coloration*: Specimens teneral. Reddish brown, with pronotum slightly paler. Head reddish brown, with small darker areas posterior to eyes. Pronotum yellowish brown, darker on disc. Elytra reddish brown. Head appendages yellowish red, legs reddish, distally darker, especially metathoracic legs (Fig. 9).

*Surface sculpture*: Submatt dorsally. Head with fine, sparse punctation (spaces between punctures 2–3 times size of punctures), only with some larger punctures between eyes; diameter of punctures smaller than diameter of cells of microreticulation. Pronotum and elytra with very fine, sparse punctation. Pronotum and elytra with rather strongly impressed microreticulation. Head with microreticulation stronger. Metaventrite and metacoxae distinctly microreticulate, metacoxal plates with longitudinal strioles and transverse wrinkles. Abdominal ventrites with distinct microreticulation, strioles, and very fine, sparse punctation.

*Structures*: Pronotum with narrow lateral bead. Its lateral sides with inconspicuous, shallow longitudinal impressions. Base of prosternum and neck of prosternal process with distinct ridge, slightly rounded anteriorly. Blade of prosternal process lanceolate, relatively broad, slightly convex, with distinct lateral bead and few setae. Abdominal ventrite 6 rounded.

*Male*: Antennae simple (Fig. 9). Protarsomere 4 with large, thick, strongly curved anterolateral hook-like seta. Protarsomere 5 long and narrow, with anterior band of ca 40 and posterior row of 6 relatively long, thin setae (Fig. 33D). Median lobe in lateral view slightly curved, broad, with apex bent downwards, not distinctly thickened, slightly angulate; in ventral view, almost subparallel and distally distinctly narrowed to apex, apex roundly truncate, of shape of a baby pacifier. Paramere slightly concave on dorsal side, with long, dense dorsal setae: subdistal setae strong and dense, setae in middle part shorter and sparser, proximal setae longer and stronger than subdistal ones (Fig. 33A–C). Abdominal ventrite 6 with 9–10 lateral striae on each side.

*Female*: Unknown.

#### Affinities.

*Exocelinapiusi* sp. n. is similar to *E.messeri* and, especially, to *E.pseudofume* sp. n. in body shape, coloration, and dorsal punctation and microreticulation, but differs from them in presence of narrow pronotal bead and shape of the median lobe.

#### Distribution.

Papua New Guinea: East Sepik Province (Fig. 50).

#### Etymology.

The species is named for Pius, a local collector. The species name is a noun in the genitive case.

### 17. Exocelina pseudofume

Taxon classificationAnimaliaColeopteraDytiscidae

Shaverdo & Balke sp. n.

http://zoobank.org/CDB94F20-4781-43BE-8079-521A5E8E6B74

Figs 7 , 32

Exocelina fume _New_Guinea_MB 4169: Toussaint et al. 2015: supplementary figs S1–S2, table S3, and information S5–S6.

#### Type locality.

Papua New Guinea: Madang Province, Wannang, 05°15.458'S, 145°02.389'E, 270 m a.s.l.

#### Type material studied.

*Holotype*: male “Papua New Guinea: Madang, Wannang, 270m 31.x.2008, 05.15.458S 145.02.389E, Posman, (PNG187)” (ZSM). *Paratypes*: 6 males, 1 female with the same label as the holotype, one of males additionally with “M.Balke 4169” [green] (NHMW, ZSM).

#### Description.

*Body size and form*: Beetle medium-sized: TL-H 3.3–3.6 mm, TL 3.6–3.95 mm, MW 1.8–2.0 mm (holotype: TL-H 3.3 mm, TL 3.6 mm, MW 1.9 mm), with oblong-oval habitus.

*Coloration*: Reddish to reddish brown, with head and pronotum paler. Head yellowish red to reddish brown, with small darker areas posterior to eyes. Pronotum yellowish red to reddish brown, darker (to brown) on disc. Elytra reddish brown to brown, sometimes with narrow yellowish or reddish sutural lines. Head appendages and legs yellowish red, legs distally darker, especially metathoracic legs (Fig. 7). All specimens are slightly teneral, therefore, the species coloration might be more darker.

*Surface sculpture*: Shiny dorsally. Head with rather dense punctation (spaces between punctures 1–2 times size of punctures), evidently finer and sparser anteriorly; diameter of punctures smaller than diameter of cells of microreticulation or equal to it. Pronotum and elytra with very distinct punctation, sparser and slightly finer than on head. Pronotum and elytra with weakly impressed microreticulation. Head with microreticulation stronger. Metaventrite and metacoxae distinctly microreticulate, metacoxal plates with longitudinal strioles and transverse wrinkles. Abdominal ventrites with distinct microreticulation, strioles, and very fine sparse punctation.

*Structures*: Pronotum without lateral bead. Base of prosternum and neck of prosternal process with distinct ridge, slightly rounded anteriorly. Blade of prosternal process lanceolate, relatively narrow, slightly convex, with distinct lateral bead and few setae. Abdominal ventrite 6 rounded.

*Male*: Antennae simple (Fig. 7). Protarsomere 4 with anterolateral seta thin, long, smaller than more laterally situated large setae, slightly curved downwards. Protarsomere 5 long and narrow, with anterior band of ca 40 and posterior row of 10 relatively long setae (Fig. 32D). Median lobe in lateral view slightly curved, its apex strongly bent downwards, with visible angle on dorsal side; in ventral view, almost subparallel and distally narrowed to truncate apex. Paramere slightly concave on dorsal side and with long, dense, thin setae, situated along dorsal margin: subdistal setae denser than setae in middle and proximal parts (Fig. 32A–C). Abdominal ventrite 6 with 5–8 lateral striae on each side.

*Female*: Without evident differences in external morphology from males, except for not modified protarsi and abdominal ventrite 6 without striae.

#### Affinities.

*Exocelinapseudofume* sp. n. is similar to *E.messeri* and *E.keki* sp. n. but it has distinctly broader and more oval habitus, shinier dorsal surface due to weaker microreticulation, as well as median lobe of different shape: thicker, with apex broader and curved downwards, with visible angle on dorsal side in lateral view. The shape of median lobe is similar to that of *E.fume*.

#### Distribution.

Papua New Guinea: Madang (Fig. 50).

#### Etymology.

The species is named “pseudofume” because shape of its median lobe remains that of *E.fume*. The name is a noun in the nominative singular standing in apposition.

### 18. Exocelina pseudopusilla

Taxon classificationAnimaliaColeopteraDytiscidae

Shaverdo & Balke sp. n.

http://zoobank.org/D1246773-6373-4A1D-AF63-B8BFBCCE69D1

Figs 18 , 42

#### Type locality.

Papua New Guinea: Simbu Province, Mount Wilhelm, 05°43'15.145"S, 145°16'10.0927"E, 1,200 m a.s.l.

#### Type material.

*Holotype*: male “Ibisca Niugini, PNG 1–3.xi.2012 Mount Wilhelm 1200 m -5,720873833 145,2694702 MW1200 / P1611 Vial 16950” (MNHN). *Paratypes*: 1 male “Ibisca Niugini, PNG 27–29.x.2012 Mount Wilhelm 1200m -5,720873833 145,2694702 MW1200 / P1513 Vial 09099” (IECA). 1 male “Ibisca Niugini, PNG 27–29.x.2012 Mount Wilhelm 1200m”, “-5,720873833 145,2694702 FIT-MW1200-E-2/8-d03 / Plot 5 / P1521 Vial 17210” (NHMW). 1 female “Ibisca Niugini, PNG 27–29.x.2012 Mount Wilhelm 1200m -5,720873833 145,2694702 MW1200 / P1553 Vial 09007” (ZSM). 1 female “Ibisca Niugini, PNG 27–29.x.2012 Mount Wilhelm 1200m -5,720873833 145,2694702 MW1200 / P1561 Vial 16873” (ZSM). 1 female “Ibisca Niugini, PNG 28–30.x.2012 Mount Wilhelm 1200m -5,720873833 145,2694702 MW1200 / P1601 Vial 17313” (ZSM). 1 female “Ibisca Niugini, PNG 28–30.x.2012 Mount Wilhelm 1200m -5,720873833 145,2694702 MW1200 / P1633 Vial 18848” (MNHN). 2 females “Ibisca Niugini, PNG 28–30.x.2012 Mount Wilhelm 1200m -5,720873833 145,2694702 MW1200 / P1609 Vial 18855” (BMNH, ZSM). 1 female “Ibisca Niugini, PNG 28–30.x.2012 Mount Wilhelm 1200m -5,720873833 145,2694702 MW1200 / P1633 Vial 18848” (ZSM). 1 female “Ibisca Niugini, PNG 30.x.-1.xi.2012 Mount Wilhelm 1200m -5,720873833 145,2694702 MW1200 / P1634 Vial 17605” (ZSM). 1 female “Ibisca Niugini, PNG 27–29.x.2012 Mount Wilhelm 1200m”, “-5,720873833 145,2694702 FIT-MW1200-G-2/8-d03 / Plot 7 / P1537 Vial 17367” (ZSM). 1 female “Ibisca Niugini, PNG 28–30.x.2012 Mount Wilhelm 1200m”, “-5,720873833 145,2694702 FIT-MW1200-S-2/8-d04 / Plot 19 / P1633 Vial 18847-CODYTI” (ANIC).

#### Description.

*Body size and form*: Beetle small: TL-H 3.25–3.55 mm, TL 3.65–3.95 mm, MW 1.8–1.85 mm (holotype: TL-H 3.5 mm, TL 3.85 mm, MW 2.0 mm), with oblong to oblong-oval habitus.

*Coloration*: Reddish brown to dark brown, with head and pronotum paler. Head reddish to reddish brown, with small darker areas posterior to eyes. Pronotum reddish to reddish brown, with dark brown disc. Elytra brown to dark brown, with narrow reddish sutural lines. Head appendages yellowish red, legs reddish, distally darker, especially metathoracic legs (Fig. 18). Teneral specimens paler.

*Surface sculpture*: Submatt dorsally. Head with dense, coarse punctation (no spaces between punctures or spaces of equal size of punctures), evidently finer and sparser anteriorly; diameter of punctures equal to or larger than diameter of cells of microreticulation. Pronotum and elytra with dense and coarse punctation, sparser and finer than on head. Pronotum and elytra with rather strongly impressed microreticulation. Head with microreticulation stronger. Metaventrite and metacoxae distinctly microreticulate, metacoxal plates with longitudinal strioles and transverse wrinkles. Abdominal ventrites with distinct microreticulation, strioles, and fine sparse punctation.

*Structures*: Pronotum with narrow lateral bead. Its lateral sides with distinct longitudinal impressions. Base of prosternum and neck of prosternal process with distinct ridge, slightly rounded anteriorly. Blade of prosternal process lanceolate, relatively broad, slightly convex, with distinct lateral bead and few setae. Abdominal ventrite 6 slightly truncate or very slightly concave.

*Male*: Antennae simple (Fig. 18). Protarsomere 4 with anterior angle slightly expanded, with large, thick, strongly curved anterolateral hook-like seta. Protarsomere 5 long and narrow, with anterior band of ca 40 and posterior row of 13 relatively long, thin setae (Fig. 42D). Median lobe in lateral view simple, slightly curved; in ventral view, evenly tapering to broadly pointed apex, side of apex slightly thickened. Paramere slightly concave on dorsal side and with dense setae on subdistal part; proximal setae finer (Fig. 42A–C). Abdominal ventrite 6 with 9–10 lateral striae on each side.

*Female*: Without evident differences in external morphology from males, except for not modified protarsi and abdominal ventrite 6 without striae.

#### Affinities.

*Exocelinapseudopusilla* sp. n. is similar to *E.pusilla* sp. n. but larger, more elongate, with denser and coarser dorsal punctation and microreticulation and differently shaped median lobe. See also under *E.cyclops* sp. n.

**Distribution and note on taxonomy.** Papua New Guinea: Simbu Province (Fig. 50). So far, this species is known only from the Mount Wilhelm, where it occurs at the high altitudes (1200 m), whilst *E.pusilla* sp. n. is also known from the Mount Wilhelm but only from 200 m. We consider the specimens from 1200 m as a distinct species (not belonging to *E.pusilla* sp. n.) because of the morphological differences mentioned above and because no intermediate forms were found. We realize a possibility that they might be just a larger, more elongate, and more strongly punctured and reticulated form of *E.pusilla* sp. n. adapted to the high altitudes. However, based on the present material, we cannot confirm it. For that, further morphological and molecular studies and more material, including one from intermediate altitudes, are requited.

#### Etymology.

The species was mistaken for *E.pusilla* sp. n. due to their similarity. The name is a noun in the nominative singular standing in apposition.

### 19. Exocelina pusilla

Taxon classificationAnimaliaColeopteraDytiscidae

Shaverdo & Balke sp. n.

http://zoobank.org/EA2F0829-7152-4555-8B5C-DE2184A07ADB

Figs 10 , 35

Exocelina undescribed sp. MB1364: Toussaint et al. 2014: supplementary figs 1–4, table 2; Toussaint et al. 2015: supplementary figs S1–S2, table S3.
Exocelina pusilla _New_Guinea_MB1364: Toussaint et al. 2015: supplementary information S5–S6.

#### Type locality.

Papua New Guinea: Madang Province, Akameku-Brahmin, Bismarck Range, 05°47.03'S, 145°24.13'E, 250–500 m a.s.l.

#### Type material.

*Holotype*: male “Papua New Guinea: Madang, Akameku-Brahmin, Bismarck Range, 250–500m, 25.xi.2006, nr 05.47.026S 145.24.131E, Balke & Kinibel (PNG 115)”, “M.Balke 1364” [green] (ZSM). *Paratypes*: **Madang**: 3 males, 31 exs. with the same label as the holotype (NHMW, ZSM). **Simbu**: 1 male “Ibisca Niugini, PNG 9–11.xi.2012 Mount Wilhelm 200m”, “-5,739897251 145,3297424 FIT-MW200-P-8/8-d16 / Plot 16 / P0835 Vial 14281-CODYTI” (ZSM). 2 females “Ibisca Niugini, PNG 11.ii-11.iv.2012 Mount Wilhelm 200m -5,739897251 145,3297424 MW0200 / P0760 Vial 07137” (ZSM). 1 female “Ibisca Niugini, PNG 2–4.xi.2012 Mount Wilhelm 200m -5,739897251 145,3297424 MW0200 / P0768 Vial 06033” (ZSM).

#### Description.

*Body size and form*: Beetle small: TL-H 2.95–3.25 mm, TL 3.2–3.6 mm, MW 1.65–1.85 mm (holotype: TL-H 2.95 mm, TL 3.25 mm, MW 1.65 mm), with oblong-oval habitus.

*Coloration*: Reddish to reddish brown, with head and pronotum paler. Head yellowish red to reddish, with small darker areas posterior to eyes. Pronotum yellowish red to reddish, with small brownish area on disc. Elytra reddish brown to brown, with narrow reddish sutural lines. Head appendages yellowish red, legs reddish, distally darker, especially metathoracic legs (Fig. 10). Teneral specimens paler.

*Surface sculpture*: Shiny dorsally. Head with dense, coarse punctation (no spaces between punctures or spaces 2 times size of punctures), evidently finer and sparser anteriorly; diameter of punctures equal to or larger than diameter of cells of microreticulation. Pronotum and elytra with dense and coarse punctation, sparser and finer than on head. Pronotum and elytra with weakly impressed microreticulation. Head with microreticulation stronger. Metaventrite and metacoxae distinctly microreticulate, metacoxal plates with longitudinal strioles and transverse wrinkles. Abdominal ventrites with distinct microreticulation, strioles, and fine sparse punctation.

*Structures*: Pronotum with narrow lateral bead. Its lateral sides with longitudinal impressions. Base of prosternum and neck of prosternal process with distinct ridge, rounded anteriorly. Blade of prosternal process lanceolate, relatively broad, slightly convex, with distinct lateral bead and few setae. Abdominal ventrite 6 slightly truncate or very slightly concave.

*Male*: Antennae simple (Fig. 10). Protarsomere 4 with anterior angle slightly expanded and large, thick, strongly curved anterolateral hook-like seta. Protarsomere 5 long and narrow, with anterior band of ca 40 and posterior row of 12 relatively long, thin setae (Fig. 35D). Median lobe in lateral view simple, slightly curved; in ventral view, evenly tapering to broadly pointed apex, side of apex slightly thickened. Paramere slightly concave on dorsal side and with dense setae on subdistal part; proximal setae finer and much sparser (Fig. 35A–C). Abdominal ventrite 6 with 5–10 lateral striae on each side.

*Female*: Without evident differences in external morphology from males, except for not modified protarsi and abdominal ventrite 6 without striae.

#### Affinities.

*Exocelinapusilla* sp. n. is very similar to *E.cyclops* sp. n. in body shape, coloration, and surface sculpture but differs in having distinctly thinner and distally narrowed median lobe. It is also similar to *E.adelbertensis* sp. n., *E.bewani* sp. n., and *E.sumokedi* sp. n. but has coarser dorsal punctation and differently shaped median lobe. See also under *E.pseudopusilla* sp. n.

#### Distribution.

Papua New Guinea: Madang and Simbu Provinces (Fig. 50).

#### Etymology.

The species name derives from the Latin “pusillus” (small, tiny) to express small size of these beetles. The species name is an adjective in the nominative singular.

### 20. Exocelina sima

Taxon classificationAnimaliaColeopteraDytiscidae

Shaverdo & Balke sp. n.

http://zoobank.org/03716131-0FB0-4A66-B212-B578D967F0DE

Figs 8 , 31

#### Type locality.

Papua New Guinea: Simbu/Eastern Highlands Province, Crater Mountain, Sera – Herowana, Sima River, ca 06°06'57.5"S, 145°03'39.4"E, 1,250 m a.s.l.

#### Type material.

*Holotype*: male “Papua New Guinea: Simbu / EHP, Crater Mountain, Sera – Herowana, Sima river, 1250m, 15IX2002, Balke & Sagata, (PNG 016)” (ZSM).

#### Description.

*Body size and form*: Beetle small: TL-H 3.6 mm, TL 4.0 mm, MW 2.0 mm, with broader, oval habitus.

*Coloration*: Reddish brown head and pronotum and piceous elytra. Head reddish in its anterior half and dark brown in posterior one. Pronotum dark brown on disc and gradually paler to yellowish red laterally. Elytra dark brown, paler laterally and almost piceous on disc. Head appendages yellowish red, legs reddish, distally darker, especially metathoracic legs (Fig. 8).

*Surface sculpture*: Shiny dorsally. Head with rather dense punctation (spaces between punctures 1–2 times size of punctures) but fine punctation; diameter of punctures smaller than diameter of cells of microreticulation. Pronotum and elytra with distinct punctation, sparser and finer punctation than on head. Pronotum and elytra with weakly impressed microreticulation. Head with microreticulation stronger. Metaventrite and metacoxae distinctly microreticulate, metacoxal plates with longitudinal strioles and transverse wrinkles. Abdominal ventrites with distinct microreticulation, strioles, and very fine sparse punctation.

*Structures*: Pronotum without lateral bead. Base of prosternum and neck of prosternal process with distinct ridge, rounded anteriorly. Blade of prosternal process lanceolate, elongate, relatively broad, slightly convex, with distinct lateral bead and few. Abdominal ventrite 6 rounded.

*Male*: Antennae simple (Fig. 8). Protarsomere 4 with anterolateral seta long and thin, equal to more laterally situated large setae, slightly curved downwards. Protarsomere 5 long and narrow, with anterior band of more than 40 and posterior row of 12 relatively long, thin setae (Fig. 31D). Median lobe in lateral view short, slightly curved, with enlarged, rounded, not bent downwards apex; in ventral view, narrow, subparallel, and with truncate apex. Paramere very slightly concave on dorsal side and with long, dense, thin setae, situated along dorsal margin: subdistal setae strong and dense, setae in middle part shorter and sparser, proximal setae long but sparser than subdistal ones (Fig. 31A–C). Abdominal ventrite 6 without lateral striae on each side, except one with setae.

*Female*: Unknown.

#### Affinities.

In absence of the pronotal bead and thin and not hook-like anterolateral seta of the male protarsomere 4, *Exocelinasima* sp. n. is similar to *E.keki* sp. n., *E.messeri*, and *E.pseudofume* sp. n. However, the species distinctly differs from them in more oval body form and more strongly expressed bicolor dorsal surface: reddish head and pronotum and piceous elytra, as well as in a characteristic shape of the median lobe and male abdominal ventrite 6 without lateral striae. The latter character is unique among New Guinea *Exocelina*.

#### Distribution.

Papua New Guinea: Simbu and Eastern Highlands Provinces, Crater Mountain. This species is known only from the type locality (Fig. 50).

#### Etymology.

The species is named after Sima River. The name is a noun in the nominative singular standing in apposition.

### 21. Exocelina simbaiensis

Taxon classificationAnimaliaColeopteraDytiscidae

Shaverdo & Balke sp. n.

http://zoobank.org/11CCE13F-F7DC-40A0-967D-C7F70EB423C2

Figs 19 , 43

Exocelina undescribed sp. MB3315: Toussaint et al. 2014: supplementary figs 1–4, table 2;
Toussaint et al. 2015: supplementary figs S1–S2, table S3.
Exocelina simbaiensis _New_Guinea_MB3315: Toussaint et al. 2015: supplementary information S5–S6.
Exocelina inengensis MB3309: Toussaint et al. 2015: supplementary figs S1–S2, table S3, and information S5–S6.

#### Type locality.

Papua New Guinea: Western Highlands Province, Simbai, Ineng River, 05°14.94'S, 144°32.82'E, 2,000 m a.s.l.

#### Type material.

*Holotype*: male “Papua New Guinea: Western Highlands, Simbai, Ineng River, 2000m, 27.ii.2007, 05.14.943S 144.32.818E, Kinibel (PNG 135)” (ZSM). *Paratypes*: 4 males, 9 females with the same label as the holotype, one male with an additional green label “M.Balke 3309” (NHMW, ZSM). 1 male, 1 female “Papua New Guinea: Western Highlands, Simbai area, 2200m, 6.iii.2007, 05.18.752S 144.31.849E, Kinibel (PNG 149)”, male additionally with “M.Balke 3316” [green] (ZSM). 2 females “Papua New Guinea: Western Highlands, Simbai area, 2500m, 8.iii.2007, 05.14.202S 144.33.651E, Kinibel (PNG 150)” (NHMW, ZSM). 1 female “M.Balke 3315” [green], “Papua New Guinea: Western Highlands, Jimi Valley, above Sendiap Station, 2000m, 6.iii.2007, 05.19.314S 144.31.266E, Kinibel (PNG 148)” (ZSM).

#### Description.

*Body size and form*: Beetle small to medium-sized: TL-H 3.3–4.15 mm, TL 3.65–4.5 mm, MW 1.8–2.15 mm (holotype: TL-H 3.9 mm, TL 4.25 mm, MW 2.0 mm), with rather oblong habitus.

*Coloration*: Dark brown to piceous, sometimes with reddish pronotum. Head dark brown to piceous, paler anteriorly. Pronotum dark brown to piceous, with reddish brown lateral sides and sometimes anteriorly and narrowly posteriorly. Elytra dark brown to piceous, sometimes with narrow reddish sutural lines. Head appendages and legs proximally yellowish red, legs distally darker, reddish (Fig. 19).

*Surface sculpture*: Matt dorsally. Head with dense punctation (no spaces between punctures or spaces 2 times size of punctures), finer and sparser anteriorly; diameter of punctures equal to diameter of cells of microreticulation. Pronotum and elytra with slightly sparser and finer punctation than on head. Head, pronotum and elytra with strongly impressed microreticulation. Metaventrite and metacoxae distinctly microreticulate, metacoxal plates with longitudinal strioles and transverse wrinkles. Abdominal ventrites with distinct microreticulation, strioles, and fine, sparse but distinct punctation.

*Structures*: Pronotum with distinct lateral bead. Its lateral sides with distinct longitudinal impressions. Base of prosternum and neck of prosternal process with distinct ridge, slightly rounded anteriorly. Blade of prosternal process lanceolate, relatively narrow, slightly convex, with distinct lateral bead and few setae. Abdominal ventrite 6 broadly rounded.

*Male*: Antennomere simple, slightly stout (Fig. 19). Protarsomere 4 with anterior angle slightly expanded, with large, thick, strongly curved anterolateral hook-like seta. Protarsomere 5 long and narrow, slightly concave ventrally, with anterior band of ca 40 and irregular posterior row of 17 relatively long setae (Fig. 43D). Median lobe in lateral view slightly curved, its apex thickened, bent downwards; in ventral view, slightly broadened medially, with broadly pointed apex. Paramere very slightly concave on dorsal side and with dense setae on subdistal part; proximal setae inconspicuous (Fig. 43A–C). Abdominal ventrite 6 with 7–12 lateral striae on each side.

*Female*: Without evident differences in external morphology from males, except for not modified protarsi and abdominal ventrite 6 without striae.

*Variability.* The specimens from Ineng River are larger: TL-H 3.5–4.15 mm; remaining specimens: TL-H 3.3–3.7 mm. In addition, the population from Ineng River shows variability in how strongly apex of the median lobe bent downwards in lateral view.

#### Affinities.

*Exocelinasimbaiensis* sp. n. is similar to *E.yoginofi* sp. n. in body form, size, and coloration, but differs from it in distinctly stronger punctation and microreticulation and in thickened apex of the median lobe.

#### Distribution.

Papua New Guinea: Western Highlands Province, near Simbai (Fig. 50).

#### Etymology.

The species is named after Simbai area. The name is an adjective in the nominative singular.

### 22. Exocelina simbaijimi

Taxon classificationAnimaliaColeopteraDytiscidae

Shaverdo & Balke sp. n.

http://zoobank.org/0B6B7A59-3532-4BE4-BAC1-7A61681B5DF1

Figs 17 , 41

Exocelina undescribed sp. MB3312: Toussaint et al. 2014: supplementary figs 1–4, table 2; Toussaint et al. 2015: supplementary figs S1–S2, table S3.
Exocelina simbaijimi _New_Guinea_MB3312: Toussaint et al. 2015: supplementary information S5–S6.

#### Type locality.

Papua New Guinea: Western Highlands Province, Simbai-Jimi, 05°16.07'S, 144°27.89'E, 1,500 m a.s.l.

#### Type material.

*Holotype*: male “Papua New Guinea: Western Highlands, Simbai - Jimi, 1500m, 2.iii.2007, 05.16.074S 144.27.886E, Kinibel (PNG 140)” (ZSM). *Paratypes*: 6 males with the same label as the holotype (NHMW, ZSM). 2 males, 3 females “Papua New Guinea: Western Highlands, Simbai, Kairong River, 1850m, 2.iii.2007, 05.14.840S 144.28.457E, Kinibel (PNG 139)”, one male with an additional green label “M.Balke 3310” (NHMW, ZSM). 3 males, 1 female “Papua New Guinea: Western Highlands, Jimi, 1500m, 2.iii.2007, 05.16.335S 144.27.930E, Kinibel (PNG 141)” (ZSM). 7 males, 1 female “Papua New Guinea: Western Highlands, Gonzsidai-Sarup, 1700m, 4.iii.2007, 05.19.060S 144.28.671E, Kinibel (PNG 144)”, one male with an additional green label “M.Balke 3312” (NHMW, ZSM). 2 males, 1 female “Papua New Guinea: Western Highlands, Kundum, 1400m, 03.III.2007, 05.16.096S 144.27.869E, Kinibel (PNG 142)” (NHMW, ZSM).

#### Additional material.

1 female “Papua New Guinea: Western Highlands, Simbai, 1800–2000m, 25.ii.2007, 05.16.330S 144.33.176E, Kinibel (PNG 133)” (ZSM). 1 female “Papua New Guinea: Western Highlands, Simbai, 1800–2000m, 1.iii.2007, 05.14.2760S 144.28.741E, Kinibel (PNG 138)” (ZSM).

#### Description.

*Body size and form*: Beetle medium-sized: TL-H 4.05–5.0 mm, TL 4.4–5.4 mm, MW 2.1–2.55 mm (holotype: TL-H 5.0 mm, TL 5.4 mm, MW 2.5 mm), with oblong-oval habitus.

*Coloration*: Piceous, with head and pronotum paler. Head dark brown to piceous, reddish brown to brown anteriorly, with small darker areas posterior to eyes. Pronotum reddish brown to brown laterally and piceous on disc. Elytra brown to piceous, sometimes with narrow reddish sutural lines. Head appendages and legs proximally reddish, legs distally darker, brown, especially metathoracic legs (Fig. 17). Teneral specimens paler.

*Surface sculpture*: Submatt dorsally. Head with rather dense, coarse punctation (spaces between punctures 1–2 times size of punctures), evidently finer and sparser anteriorly; diameter of punctures smaller than or equal to diameter of cells of microreticulation. Pronotum with distinct punctation, sparser and finer than on head. Elytra with very fine and sparse punctation. Pronotum and elytra with strongly impressed microreticulation. Head with microreticulation stronger. Metaventrite and metacoxae distinctly microreticulate, metacoxal plates with longitudinal strioles and transverse wrinkles. Abdominal ventrites with distinct microreticulation, strioles, and fine, sparse punctation.

*Structures*: Pronotum with distinct lateral bead. Its lateral sides with longitudinal impressions. Base of prosternum and neck of prosternal process with distinct ridge, slightly rounded anteriorly. Blade of prosternal process lanceolate, relatively narrow, slightly convex, and smooth, with distinct lateral bead and few lateral setae. Abdominal ventrite 6 slightly truncate or broadly rounded.

*Male*: Antennae simple (Fig. 17). Protarsomere 4 with anterior angle slightly expanded, with large, thick, strongly curved anterolateral hook-like seta. Protarsomere 5 long and narrow, slightly concave ventrally, with anterior band of more than 80 and posterior band of ca 30 relatively long setae (Fig. 41D). Median lobe in lateral view evenly broad, with rounded, slightly angulated, thickened apex; in ventral view, almost subparallel, with broadly rounded apex, with thickened margins. Paramere slightly concave on dorsal side and with long, dense, thin setae situated along dorsal margin: subdistal setae denser, proximal setae sparser, setae in middle finer (Fig. 41A–C). Abdominal ventrite 6 with 9–14 lateral striae on each side.

*Female*: Without evident differences in external morphology from males, except for not modified protarsi and abdominal ventrite 6 without striae.

#### Affinities.

*Exocelinasimbaijimi* sp. n. is similar to *E.heidiae* in body size and form but differs from it in darker coloration, more distinct dorsal punctation, broader median lobe, with more angulated apex in lateral view and more thickened margins in ventral view.

#### Distribution.

Papua New Guinea: Western Highlands Province (Fig. 50).

#### Etymology.

The species is named after Simbai-Jimi area. The name is a noun in the nominative singular standing in apposition.

### 23. Exocelina sumokedi

Taxon classificationAnimaliaColeopteraDytiscidae

Shaverdo & Balke sp. n.

http://zoobank.org/23A6509D-8E6A-4D0D-B585-0073ADE777D3

Figs 11 , 34

#### Type locality.

Papua: Puncak Regency, south from Iratoi, 03°16'48.6"S, 137°20'02.9"E, 150 m a.s.l.

#### Type material.

*Holotype*: male “Indonesia: Papua, S Iratoi, hunting camp, 150m, 28.v.2006, -3,2801742386 137,334125172346, local collectors” (MZB). *Paratypes*: 11 males, 13 females with the same label as the holotype (MZB, NHMW, ZSM). 6 males, 2 females “Indonesia: Papua, S Iratoi, river camp, 161m, 20./25.v.2006, -3,3522959 137,295029880478, local collectors” (NHMW, ZSM). 1 female “Indonesia: Papua, Rouaffer, Iratoi, hill in forest, 164m, 6.ix.2005, -3,2403086 137,3329744, local collectors” (ZSM).

#### Description.

*Body size and form*: Beetle small: TL-H 2.7–3.2 mm, TL 3.0–3.55 mm, MW 1.55–1.9 mm (holotype: TL-H 2.95 mm, TL 3.25 mm, MW 1.75 mm), with broader, oval habitus.

*Coloration*: Brownish, with head and pronotum paler. Head yellowish red to reddish brown in anterior half and brown to dark brown in posterior ones. Pronotum yellowish red to reddish brown on sides, brown to dark brown on disc. Elytra brown to dark brown, with narrow reddish sutural lines. Head appendages yellowish red, legs reddish, distally darker, especially metathoracic legs (Fig. 11). Teneral specimens paler.

*Surface sculpture*: Shiny dorsally. As in *E.pusilla* sp. n. but punctation finer and sparser and microreticulation weakly impressed.

*Structures*: Pronotum with lateral bead. Its lateral sides with shallow longitudinal impressions. Base of prosternum and neck of prosternal process with distinct ridge, rounded anteriorly. Blade of prosternal process lanceolate, relatively broad, slightly convex, with distinct lateral bead and few setae. Abdominal ventrite 6 broadly rounded or slightly truncate.

*Male*: Antennae simple (Fig. 11). Protarsomere 4 with medium-sized, thick, curved anterolateral hook-like seta. Protarsomere 5 long and narrow, with anterior row of 17 and posterior row of 6 relatively short setae (Fig. 34D). Median lobe in lateral view slightly curved, with thickened, not curved downwards apex; in ventral view, distinctly narrowed subdistally, with roundly truncate apex. Paramere slightly concave on dorsal side and with dense setae on subdistal part; proximal setae finer and sparser (Fig. 34A–C). Abdominal ventrite 6 with 5–8 lateral striae on each side.

*Female*: Without evident differences in external morphology from males, except for not modified protarsi and abdominal ventrite 6 without striae.

#### Affinities.

*Exocelinasumokedi* sp. n. is similar to *E.pusilla* sp. n. but is smaller, darker, more oval, shinier, with finer and sparser dorsal punctation and weaker microreticulation, with shallow longitudinal impressions on lateral sides of pronotum and different shape of the median lobe. The species is also similar to *E.adelbertensis* sp. n., *E.bewani* sp. n., *E.cyclops* sp. n., and *E.pseudopusilla* sp. n., see their “Affinities” and “Key”.

#### Distribution.

Papua: Puncak Regency. The species is known only from the Iratoi area (Fig. 50).

#### Etymology.

The species is named for our friend Bob Sumoked (Tomohon, Sulawesi). The species name is a noun in the genitive case.

### 24. Exocelina yoginofi

Taxon classificationAnimaliaColeopteraDytiscidae

Shaverdo & Balke sp. n.

http://zoobank.org/1F4C342B-17BB-4331-83CF-5AC202F7A881

Figs 22 , 46

Exocelina undescribed sp. MB1302: Toussaint et al. 2014: supplementary figs 1–4, table 2; Toussaint et al. 2015: supplementary figs S1–S2, table S3.
Exocelina yoginofi _New_Guinea_MB1302: Toussaint et al. 2015: supplementary information S5–S6.

#### Type locality.

Papua New Guinea: Eastern Highlands Province, Kainantu, Yoginofi, 06°21.80'S, 145°45.46'E, 1,900 m a.s.l.

#### Type material.

*Holotype*: male “Papua New Guinea: Eastern Highlands, Kainantu, Yoginofi, 1900m, 9.v.1994, 06.21.799S 145.45.463E, Balke & Sagata (PNG 55)” (ZSM). *Paratypes*: 6 males, 8 females with the same label as the holotype, one male with an additional green label “M.Balke 1302” (NHMW, ZSM). 1 male, 1 female “Papua New Guinea: Eastern Highlands, 37 km S Goroka, Hogave vill., Mt. Michael, 2179–2800m, 9.-15.vii.2009, 06.22.479S 145.15.256E, Sagata (PNG230)” (ZSM).

#### Description.

*Body size and form*: Beetle medium-sized: TL-H 3.45–4.0 mm, TL 3.85–4.45 mm, MW 1.85–2.15 mm (holotype: TL-H 3.9 mm, TL 4.3 mm, MW 2.0 mm), with oblong-oval habitus.

*Coloration*: Dark brown to piceous, with paler pronotum. Head reddish to dark brown, paler anteriorly and posteriorly. Pronotum brown to dark brown, with reddish brown lateral sides and sometimes anteriorly and narrowly posteriorly, darker on disc, sometimes to piceous. Elytra brown to piceous, sometimes with narrow reddish sutural lines. Head appendages and legs proximally yellowish red, legs distally darker, reddish brown (Fig. 22).

*Surface sculpture*: Submatt dorsally. Head with rather dense punctation (spaces between punctures 1–2 times size of punctures), finer and sparser anteriorly; diameter of punctures smaller than or equal to diameter of cells of microreticulation. Pronotum and elytra with sparser and finer punctation than on head. Head, pronotum and elytra with rather strongly impressed microreticulation. Metaventrite and metacoxae distinctly microreticulate, metacoxal plates with longitudinal strioles and transverse wrinkles. Abdominal ventrites with distinct microreticulation, strioles, and fine, sparse but distinct punctation.

*Structures*: Pronotum with distinct lateral bead. Its lateral sides with distinct longitudinal impressions. Base of prosternum and neck of prosternal process with distinct ridge, rounded anteriorly. Blade of prosternal process lanceolate, relatively broad, slightly convex, with distinct lateral bead and few setae. Abdominal ventrite 6 rounded.

*Male*: Antennomere simple (Fig. 22). Protarsomere 4 with large, thick, strongly curved anterolateral hook-like seta. Protarsomere 5 long and narrow, with narrow anterior band of ca. 40 and posterior row of 14 relatively long setae (Fig. 46D). Median lobe in lateral view slightly curved, with apex dully pointed, slightly bent downwards; in ventral view, broadened subdistally, with broad, rounded apex. Paramere very slightly concave on dorsal side and with dense setae on subdistal part; proximal setae inconspicuous (Fig. 46A–C). Abdominal ventrite 6 with 6–8 lateral striae on each side.

*Female*: Without evident differences in external morphology from males, except for not modified protarsi and abdominal ventrite 6 without striae.

#### Affinities.

*Exocelinayoginofi* sp. n. is similar to *E.simbaiensis* sp. n. in body form, size, and coloration, but differs from it in distinctly finer punctation and microreticulation and in having the apex of the median lobe not thickened. Also, see under *E.okapa* sp. n.

#### Distribution.

Papua New Guinea: Eastern Highlands Province (Fig. 50).

#### Etymology.

The species is named after Yoginofi Village. The name is a noun in the nominative singular standing in apposition.

### Key to species of the *Exocelinacasuarina*-group

The key is based mostly on the male characters. In many cases, females cannot be assigned to species due to similarity of their external and internal structures (for female genitalia see Figs 17a and 17b in Shaverdo et al. (2005)). Some species are rather similar in point of external morphology; therefore, in most cases the male genitalia need to be studied for reliable species identification. Numbers in brackets refer to an arrangement of the species descriptions above.

**Table:** 

1	Pronotum without lateral bead	**2**
–	Pronotum with lateral bead	**8**
2	Male protarsomere 4 with anterolateral seta hook-like, large, strongly curved	**3**
–	Male protarsomere 4 with anterolateral seta thin, long, equal or smaller than more laterally situated large setae, slightly curved	**5**
3	Median lobe not or slightly narrowed before truncate or slightly concave apex in ventral view (Fig. 26A)	(4) ***casuarina***
–	Median lobe distinctly narrowed before truncate apex in ventral view (Figs 27A, 28A)	**4**
4	Apex of median lobe curved downwards, with visible angle on dorsal side in lateral view (Fig. 27B). Dorsal punctation coarser, microreticulation more strongly impressed	(7) ***fume***
–	Apex of median lobe not or only slightly curved downwards in lateral view (Fig. 28B). Dorsal punctation distinctly finer, microreticulation less strongly impressed	(9) ***ibalimi***
5	Beetle more oval, broader, with pronotum reddish brown and elytra piceous (Fig. 8). Median lobe shorter, almost parallel-sided in ventral view, with distinctly rounded apex in lateral view (Fig. 31A, B)	(20) ***sima***
–	Beetle elongate, narrower, with reddish to reddish brown dorsal coloration (Figs 5–7). Median lobe different	**6**
6	Dorsal punctation almost invisible on elytra (Fig. 5). Median lobe narrowed to slightly rounded, broad apex in ventral view (Fig. 29A). Ventral setae of male protarsomere 5 much more numerous, usually not divided into two rows/bands (Fig. 29D)	(14) ***messeri***
–	Dorsal punctation distinct on elytra (Figs 6, 7). Median lobe different. Ventral setae of male protarsomere 5 much less numerous, clearly divided into anterior band and posterior row	**7**
7	Dorsal microreticulation more strongly impressed (Fig. 6), beetle submatt. Apex of median lobe narrow and rounded in ventral view and not curved downwards in lateral view (Fig. 30A, B).	(10) ***keki***
–	Dorsal microreticulation less strongly impressed (Fig. 7), beetle shinier. Apex of median lobe broad and truncate in ventral view and strongly curved downwards in lateral view (Fig. 32 A, B)	(17) ***pseudofume***
8	Beetle reddish brown, more oval. Usually smaller, TL-H < 3.6 mm	**9**
–	Beetle reddish brown to piceous, elongate, oblong-oval. Usually larger, TL-H > 4.0 mm; if smaller, see below	**14**
9	Beetle larger, TL-H 3.5–3.6 mm, with finer and sparser dorsal punctation and weaker microreticulation. Median lobe as in Fig. 33A, B	(16) ***piusi***
–	Beetle smaller, TL-H 2.7–3.25 mm, usually with distinctly coarser dorsal punctation and sometimes, stronger microreticulation. Median lobe different (e.g., Fig. 34)	**10**
10	Median lobe with thinner apex in lateral view, apex narrowed to tip in ventral view (Fig. 35B, A). Male protarsomere 4 with larger anterolateral hook-like seta; anterior setae of male protarsomere 5 more numerous (Fig. 35D)	(19) ***pusilla***
–	Median lobe with apex thickened in lateral view, apex not narrowed to tip, broad, differently truncate in ventral view (e.g., Fig. 36B, A). Male protarsomere 4 with smaller anterolateral hook-like seta; anterior setae of male protarsomere 5 less numerous (e.g., Fig. 36D)	**11**
11	Median lobe distinctly narrowed distally, with apex roundly truncate in ventral view (Fig. 34A)	(23) ***sumokedi***
–	Median lobe not or very slightly narrowed distally, with apex distinctly truncate or slightly concave in ventral view (e.g., Fig. 36A)	**12**
12	Apex of median lobe not curved downwards in lateral view (Fig. 36B)	(5) ***cyclops***
–	Apex of median lobe curved downwards in lateral view (Figs 37B, 38B)	**13**
13	Apex of median lobe narrower in lateral view and slightly concave in ventral view (Fig. 37A)	(3) ***bewani***
–	Apex of median lobe broader in lateral view and truncate in ventral view (Fig. 38A)	(1) ***adelbertensis***
14	Apex of median lobe strait, flatted, and thin apex in lateral view and broadly elongated, lanceolate, impressed in ventral view (Fig. 44A, B)	(13) ***menyamya***
–	Apex of median lobe of different shape, never so flatted and impressed ventrally, usually thickened in lateral view (e.g., Fig. 39A, B)	**15**
15	Median lobe evenly broad, with rounded, not curved downwards apex in lateral view (e.g., Fig. 39B)	**16**
–	Median lobe narrowed towards apex, apex pointed or slightly rounded, usually curved downwards in lateral view (e.g., Fig. 43B)	**18**
16	Beetle smaller, TL-H 3.5–4.3 mm, reddish brown to brown, with distinctly stronger dorsal punctation. Median lobe smaller and thinner (Fig. 39A, B)	(6) ***desii***
–	Beetle larger, TL-H 4.05–5.0 mm, dark brown to piceous, with dorsal punctation finer and sparser. Median lobe larger and more robust (Figs 40, 41)	**17**
17	Beetle dark brown, with elytral punctation finer. Median lobe thinner and narrower in lateral view (Fig. 40 B)	(8) ***heidiae***
–	Beetle dark brown to piceous, with elytral punctation more distinct. Median lobe thicker and broader in lateral view (Fig. 41 B)	(22) ***simbaijimi***
18	Beetle more elongate, almost parallel-sided, smaller, TL-H 3.25–4.15 mm, with strong dorsal punctation and microreticulation	**19**
–	Beetle more oval, larger, TL-H 3.45–5.5 mm, with dorsal punctation and microreticulation in some species much finer and sparser	**20**
19	Beetle smaller, TL-H 3.25–3.55 mm, reddish brown to dark brown (Fig. 18). Apex of median lobe slightly thickened, not bent downwards in lateral view (Fig. 42B)	(18) ***pseudopusilla***
–	Beetle larger, TL-H 3.3–4.15 mm, dark brown to piceous (Fig. 19). Apex of median lobe more strongly thickened, bent downwards in lateral view (Fig. 43B)	(21) ***simbaiensis***
20	Beetle smaller, TL-H 3.45–4.7 mm	**21**
–	Beetle larger, TL-H 4.8–5.5 mm	**23**
21	Apex of median lobe not bent downwards in lateral view, roundly truncate in ventral view (Fig. 45B, A). Beetle matt, with strong dorsal microreticulation (Fig. 21)	(2) ***ambua***
–	Apex of median lobe bent downwards in lateral view, rounded in ventral view (e.g., Fig. 46B, A). Beetle shiny or submatt, with dorsal microreticulation weaker (e.g., Fig. 22)	**22**
22	Beetle smaller, TL-H 3.45–4.0 mm, submatt, with distinct dorsal punctation and microreticulation (Fig. 22). Median lobe as in Fig. 46A, B	(24) ***yoginofi***
–	Beetle larger, TL-H 3.95–4.7 mm, shiny, with extremely fine, inconspicuous dorsal punctation and weak microreticulation (Fig. 23). Median lobe as in Fig. 47A, B	(15) ***okapa***
23	Dorsal punctation and microreticulation weaker (Fig. 24). Median lobe as in Fig. 48A, B	(12) ***mendiensis***
–	Dorsal punctation and microreticulation stronger (Fig. 25). Median lobe as in Fig. 49A, B	(11) ***kumulensis***

### Habitat

The studied species have the same habitat preferences as those described in Shaverdo et al. (2012). They are associated with running water, but avoid the current, i.e., their preferred microhabitats are small creeks, small and quiet backflows, puddles at the edge of streams and creeks, and other similar situations.

**Figures 15–17.**
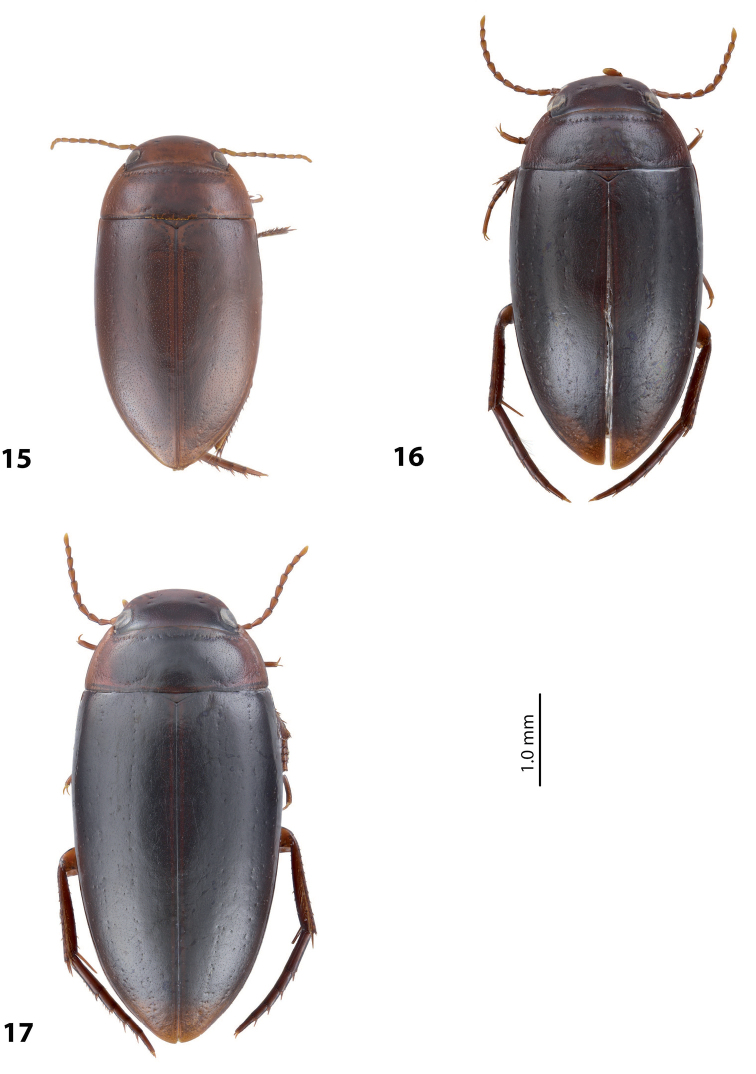
Habitus and coloration **15***Exocelinadesii* (Balke, 1999) **16***E.heidiae* (Balke, 1998) **17***E.simbaijimi* sp. n.

**Figures 18–21.**
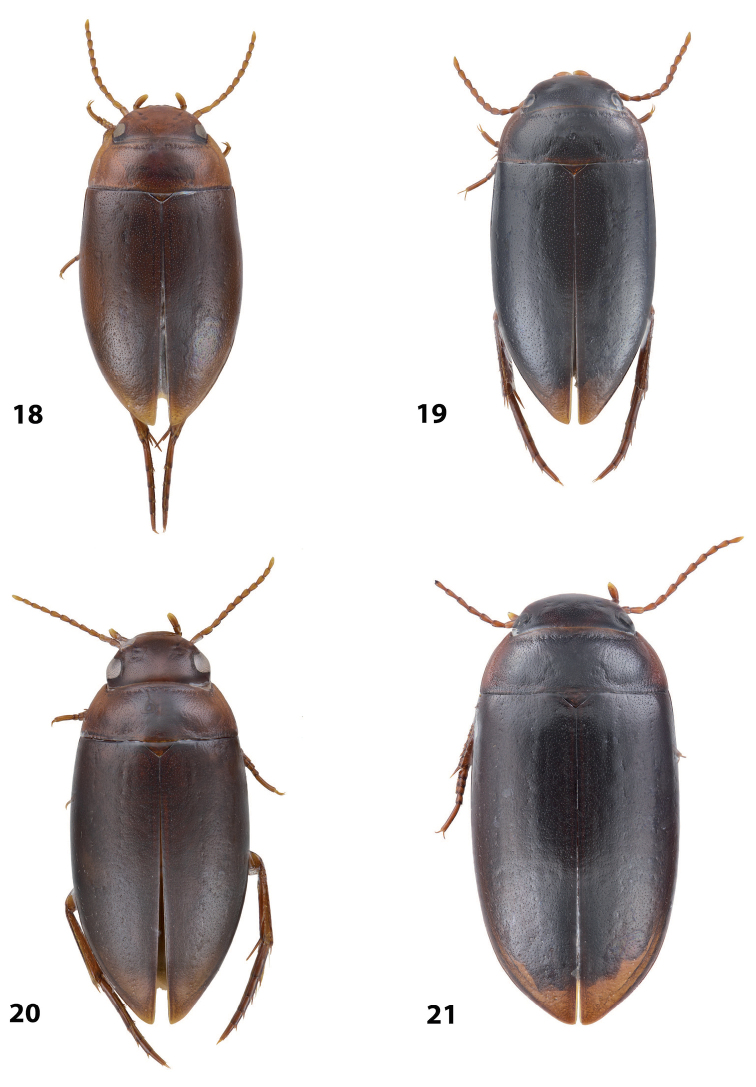
Habitus and coloration **18***Exocelinapseudopusilla* sp. n. **19***E.simbaiensis* sp. n. **20***E.menyamya* sp. n. **21***E.ambua* sp. n.

**Figures 22–25.**
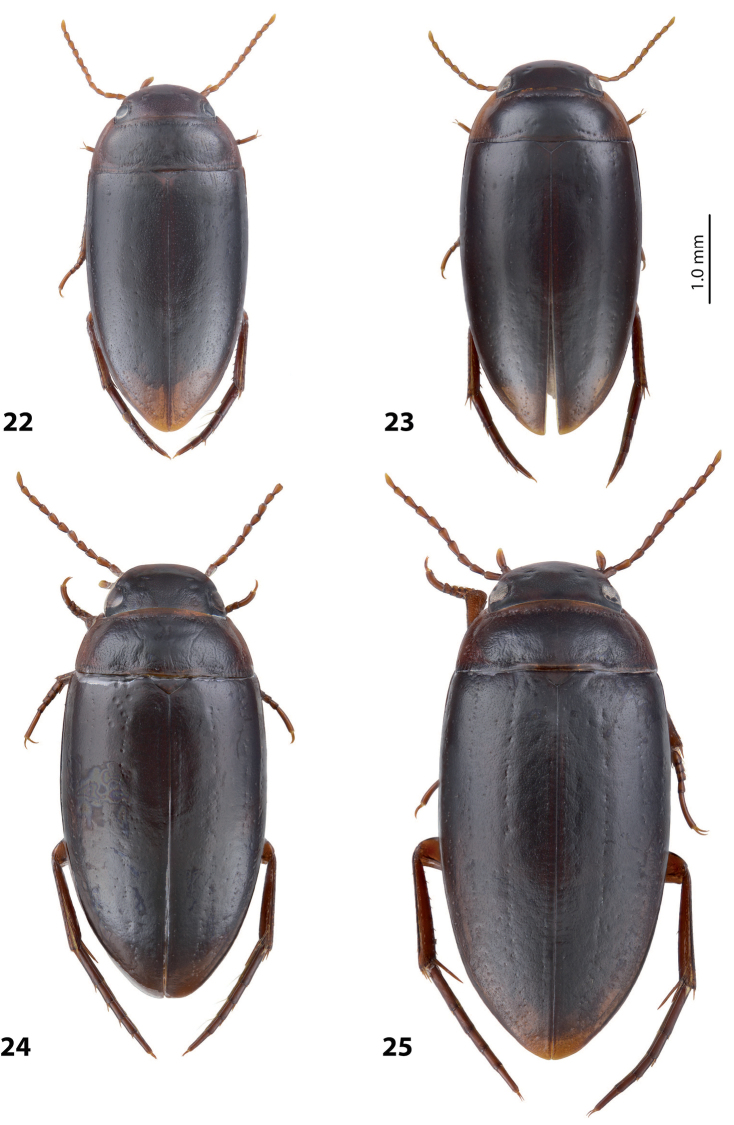
Habitus and coloration **22***Exocelinayoginofi* sp. n. **23***E.okapa* sp. n. **24***E.mendiensis* sp. n. **25***E.kumulensis* sp. n.

**Figure 26.**
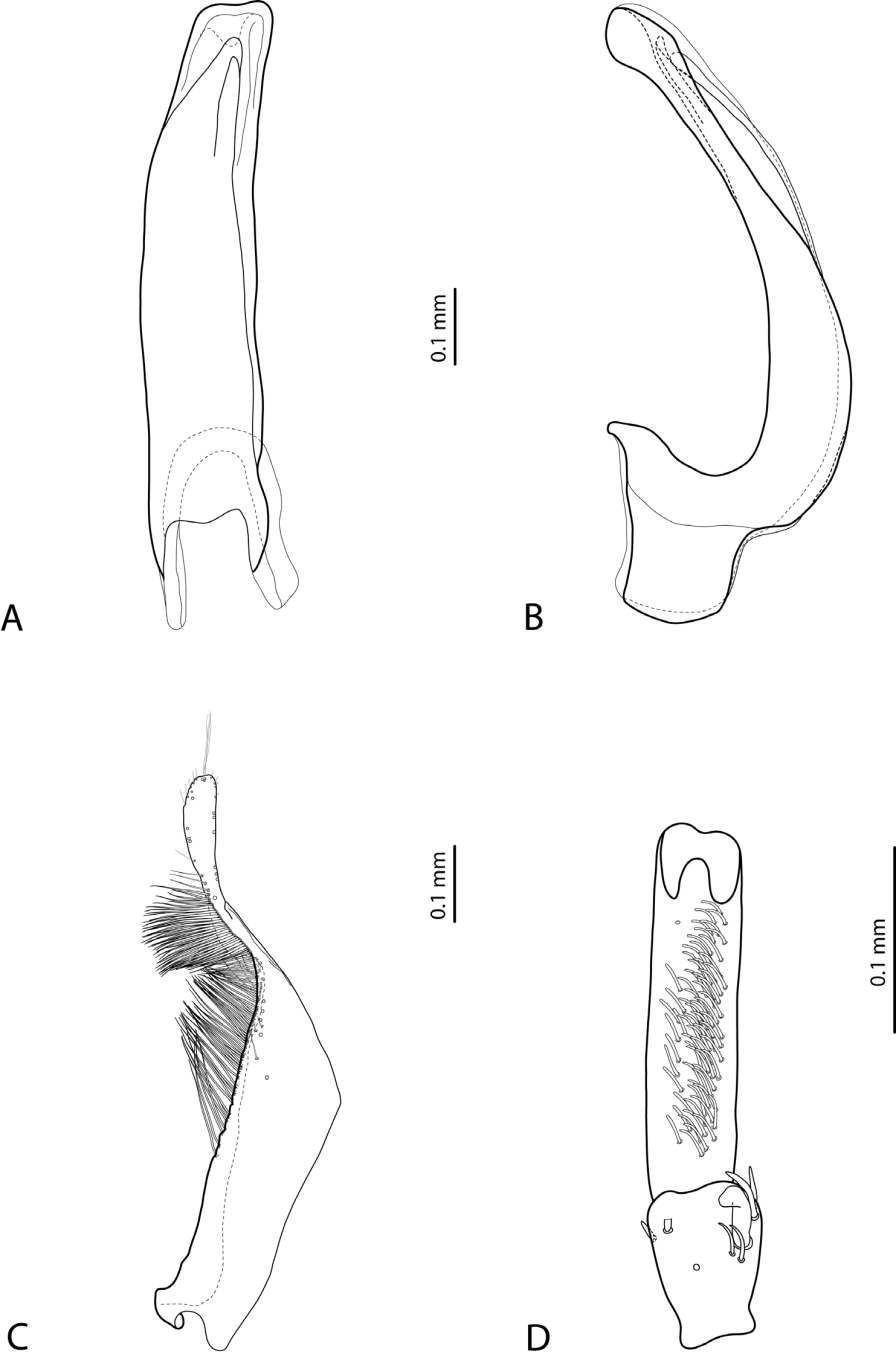
*Exocelinacasuarina* (Balke, 1998) **A** Median lobe in ventral view **B** Median lobe in lateral view **C** Paramere in external view **D** Male protarsomeres 4–5 in ventral view.

**Figures 27–28.**
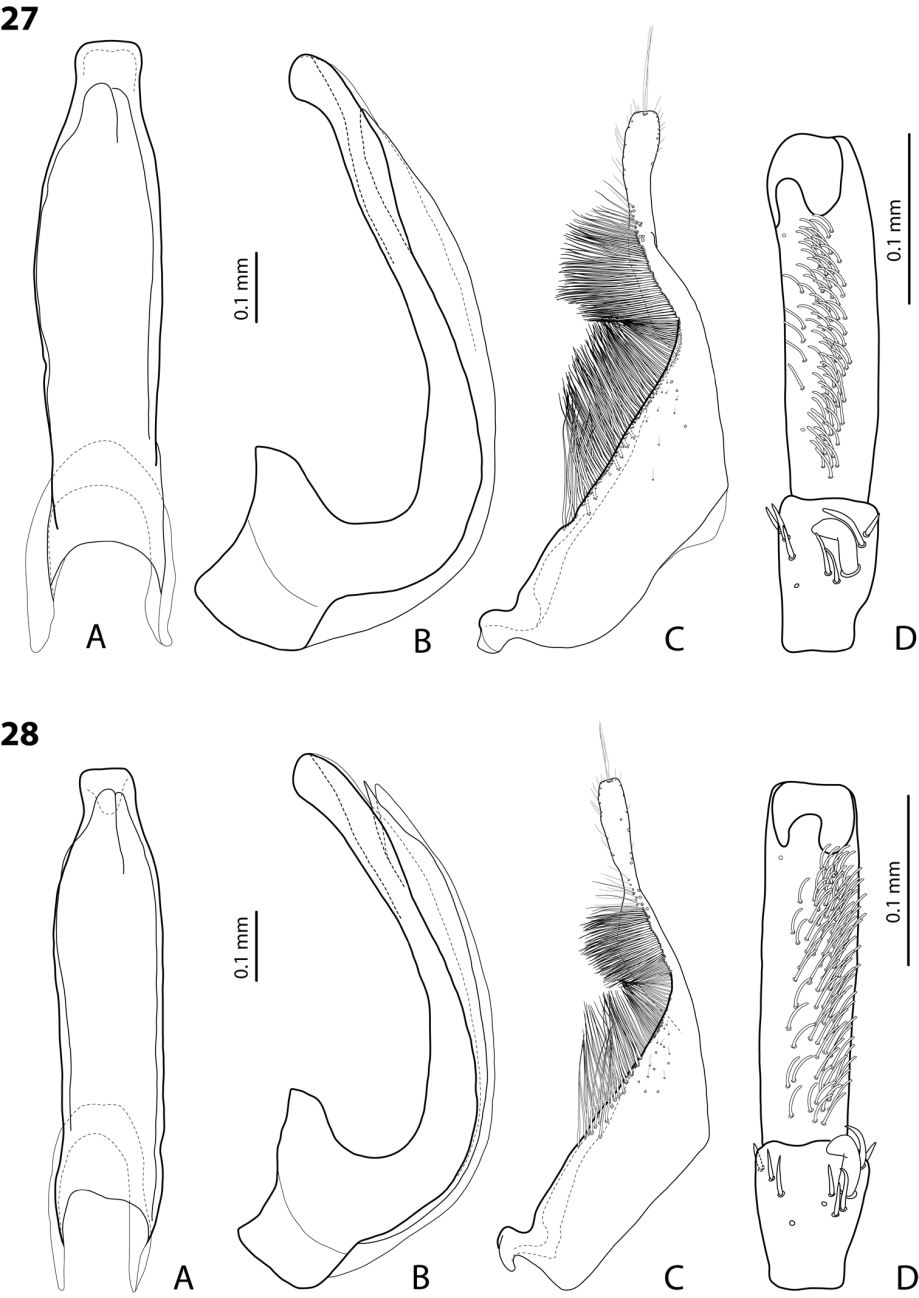
**27***Exocelinafume* (Balke, 1998) **28***E.ibalimi* sp. n. **A** Median lobe in ventral view **B** Median lobe in lateral view **C** Paramere in external view **D** Male protarsomeres 4–5 in ventral view.

**Figures 29–30.**
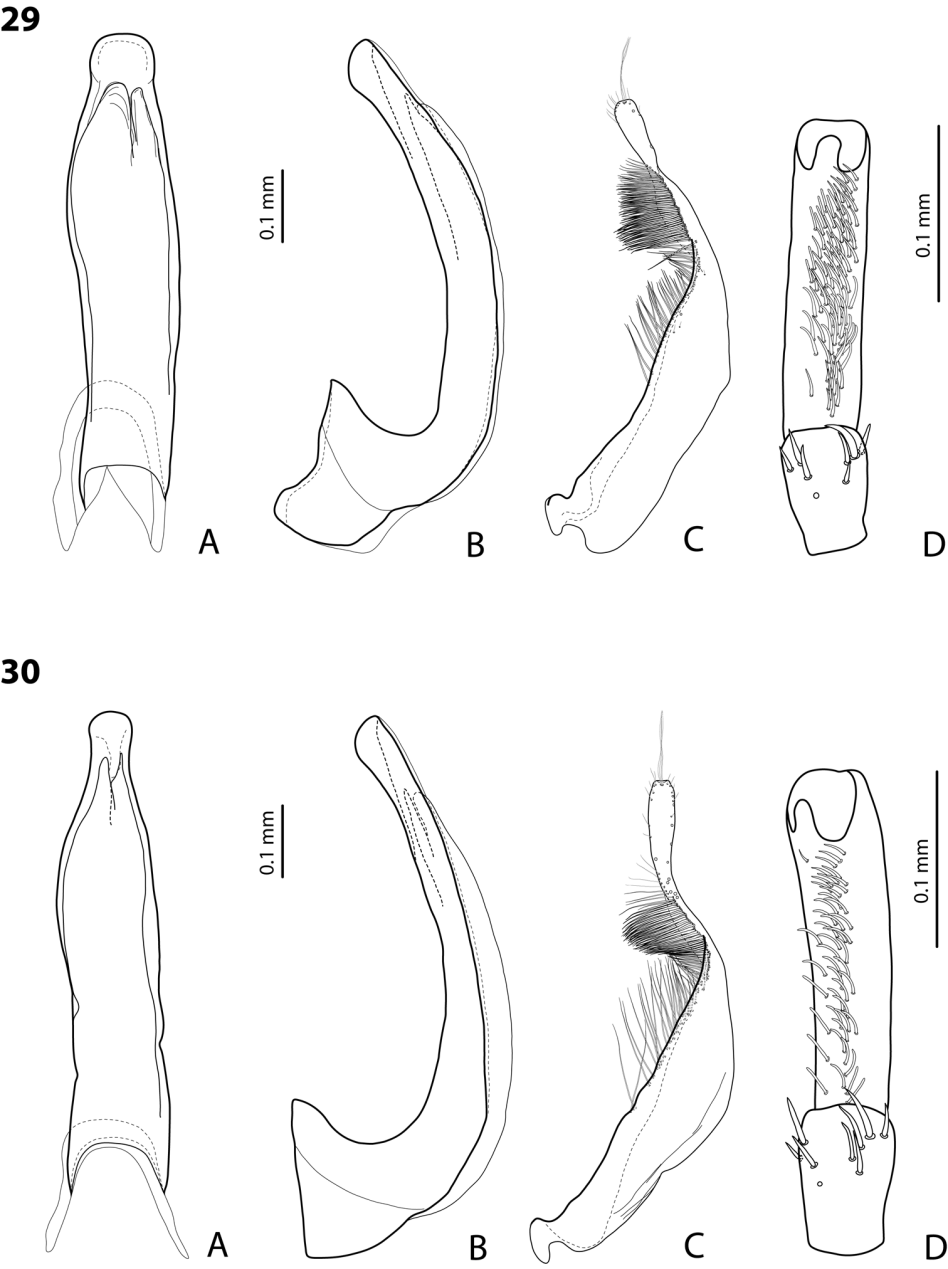
**29***Exocelinamesseri* (Balke, 1999) **30***E.keki* sp. n. **A** Median lobe in ventral view **B** Median lobe in lateral view **C** Paramere in external view **D** Male protarsomeres 4–5 in ventral view.

**Figures 31–32.**
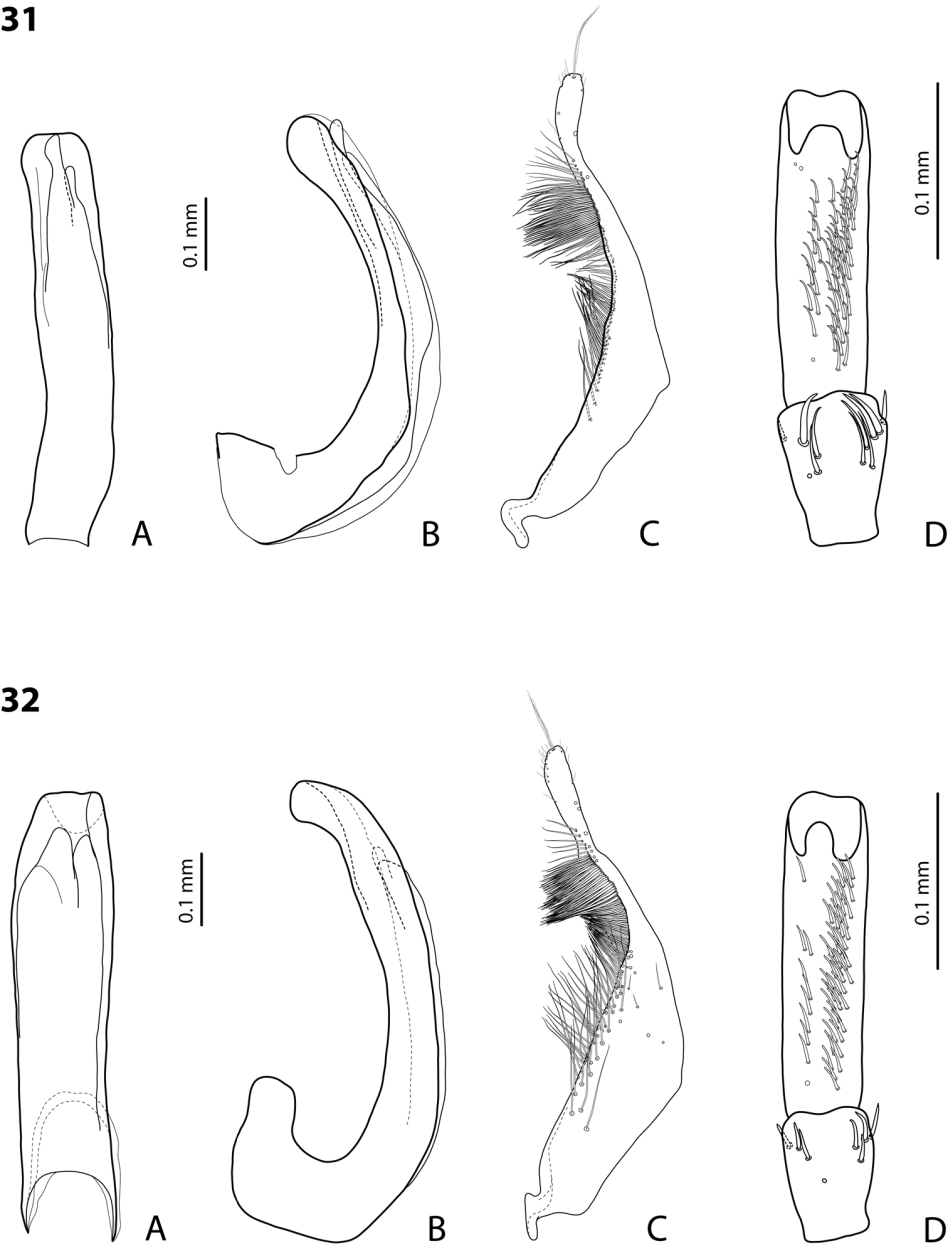
**31***Exocelinasima* sp. n. **32***E.pseudofume* sp. n. **A** Median lobe in ventral view **B** Median lobe in lateral view **C** Paramere in external view **D** Male protarsomeres 4–5 in ventral view.

**Figures 33–34.**
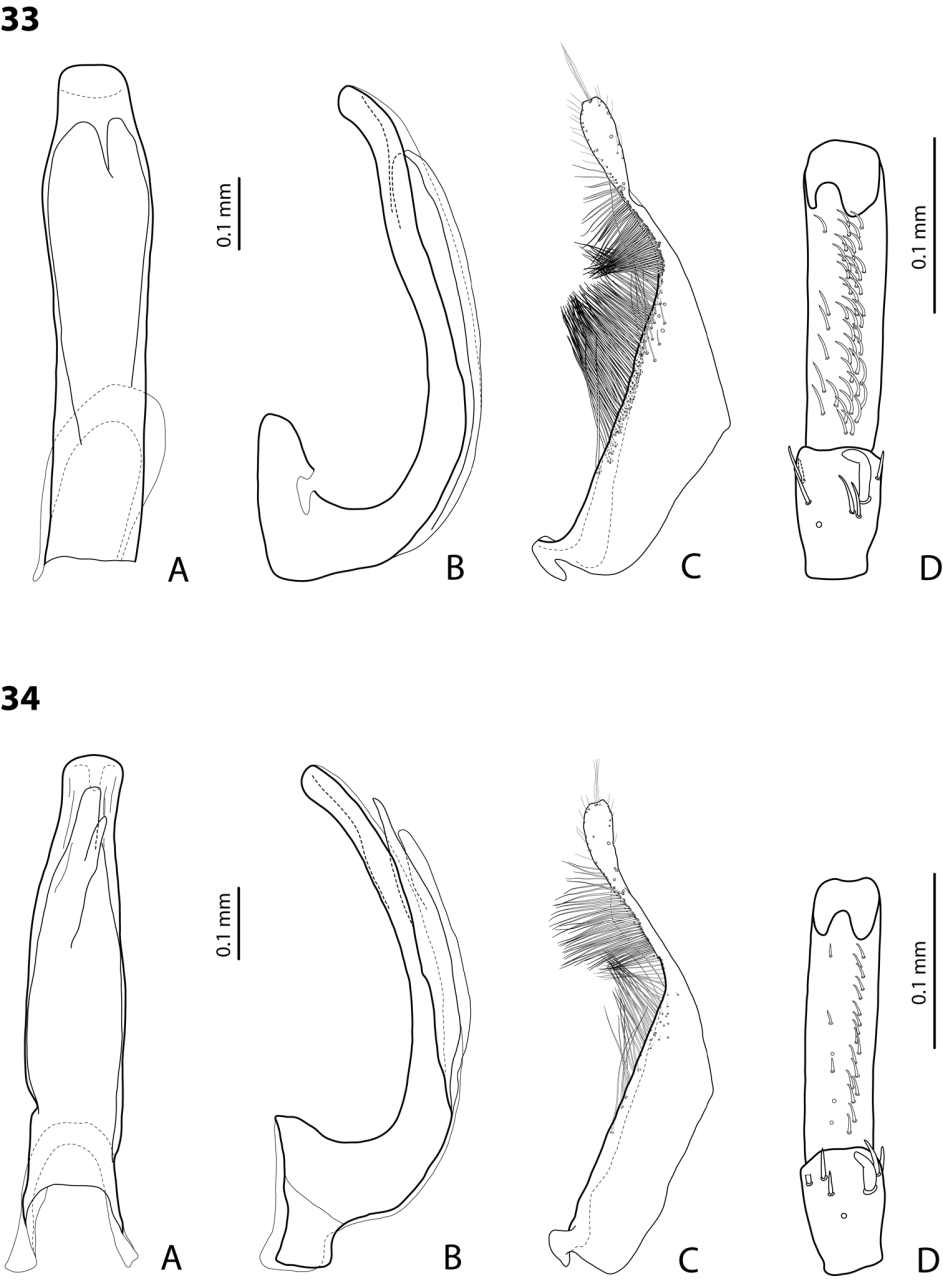
**33***Exocelinapiusi* sp. n. **34***E.sumokedi* sp. n. **A** Median lobe in ventral view **B** Median lobe in lateral view **C** Paramere in external view **D** Male protarsomeres 4–5 in ventral view.

**Figures 35–36.**
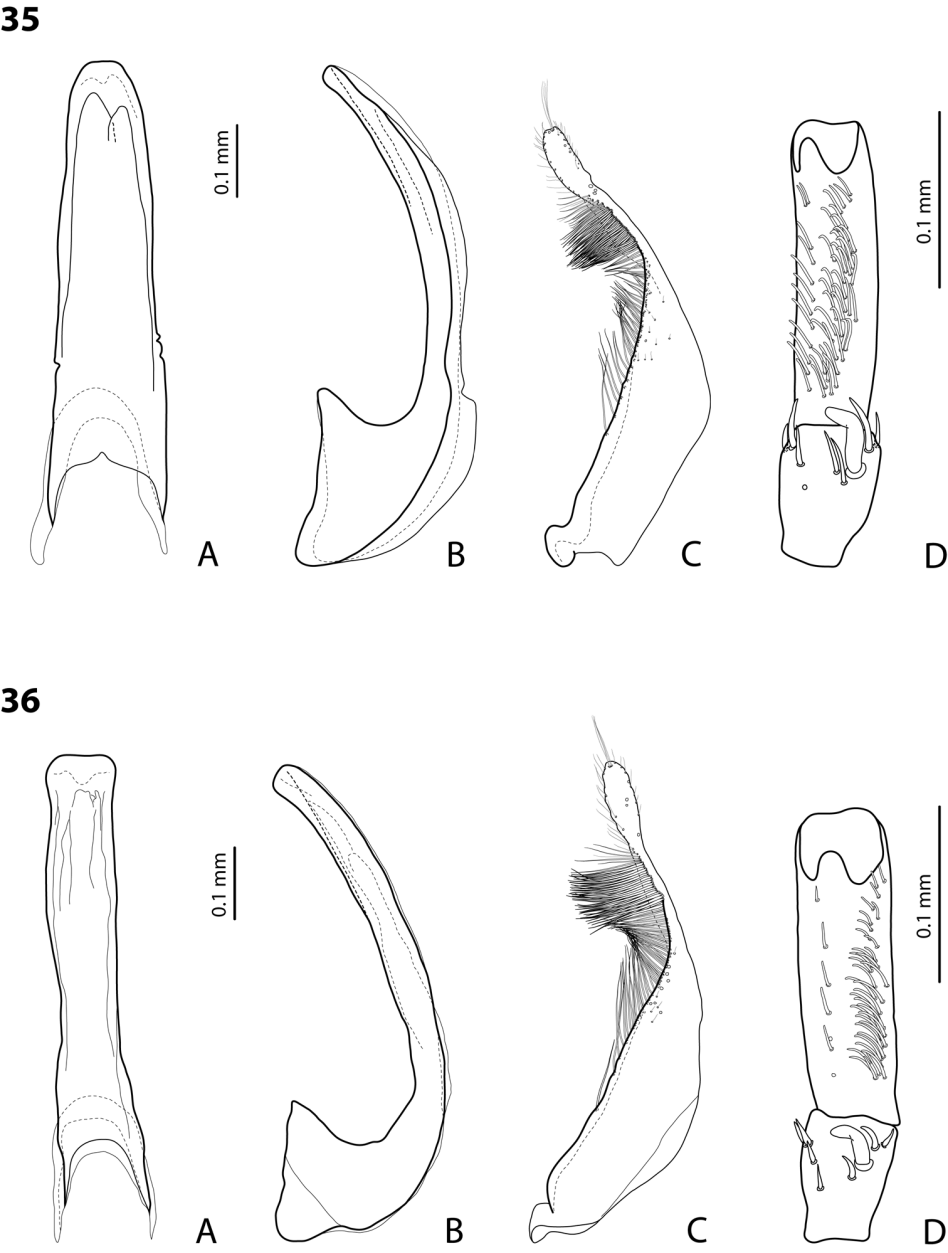
**35***Exocelinapusilla* sp. n. **36***E.cyclops* sp. n. **A** Median lobe in ventral view **B** Median lobe in lateral view **C** Paramere in external view **D** Male protarsomeres 4–5 in ventral view.

**Figures 37–38.**
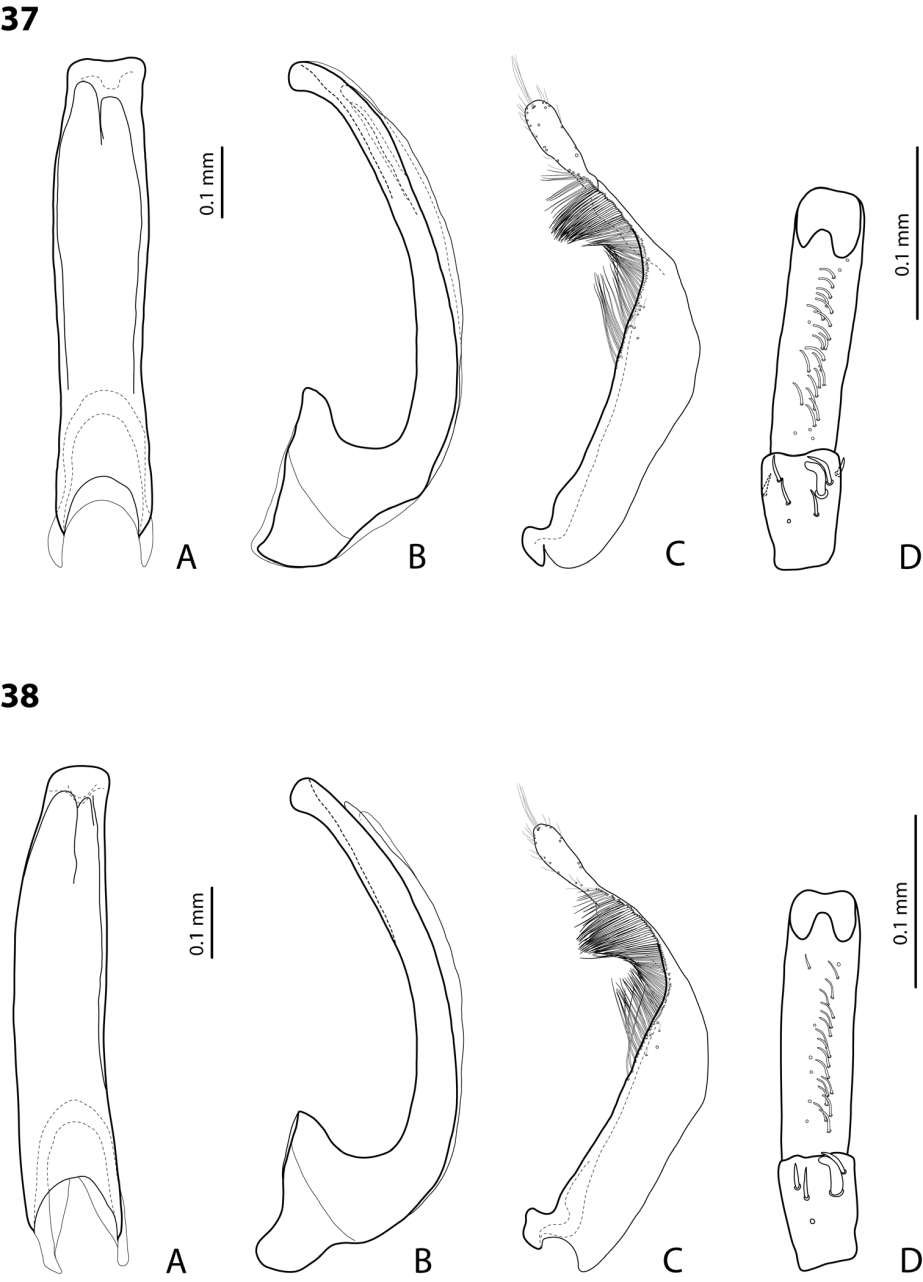
**37***Exocelinabewani* sp. n. **38***E.adelbertensis* sp. n. **A** Median lobe in ventral view **B** Median lobe in lateral view **C** Paramere in external view **D** Male protarsomeres 4–5 in ventral view.

**Figure 39.**
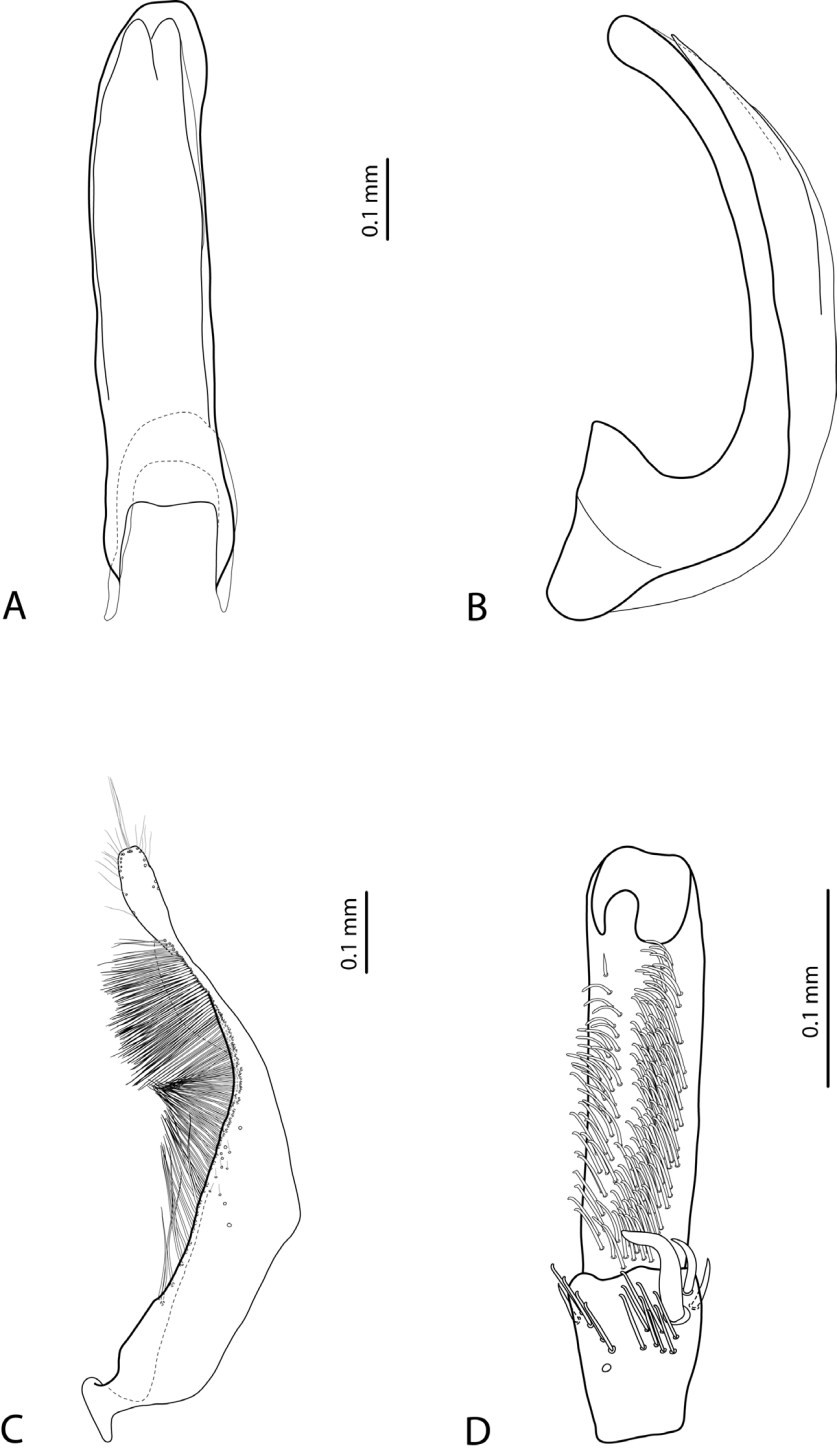
*Exocelinadesii* (Balke, 1999) **A** Median lobe in ventral view **B** Median lobe in lateral view **C** Paramere in external view **D** Male protarsomeres 4–5 in ventral view.

**Figures 40–41.**
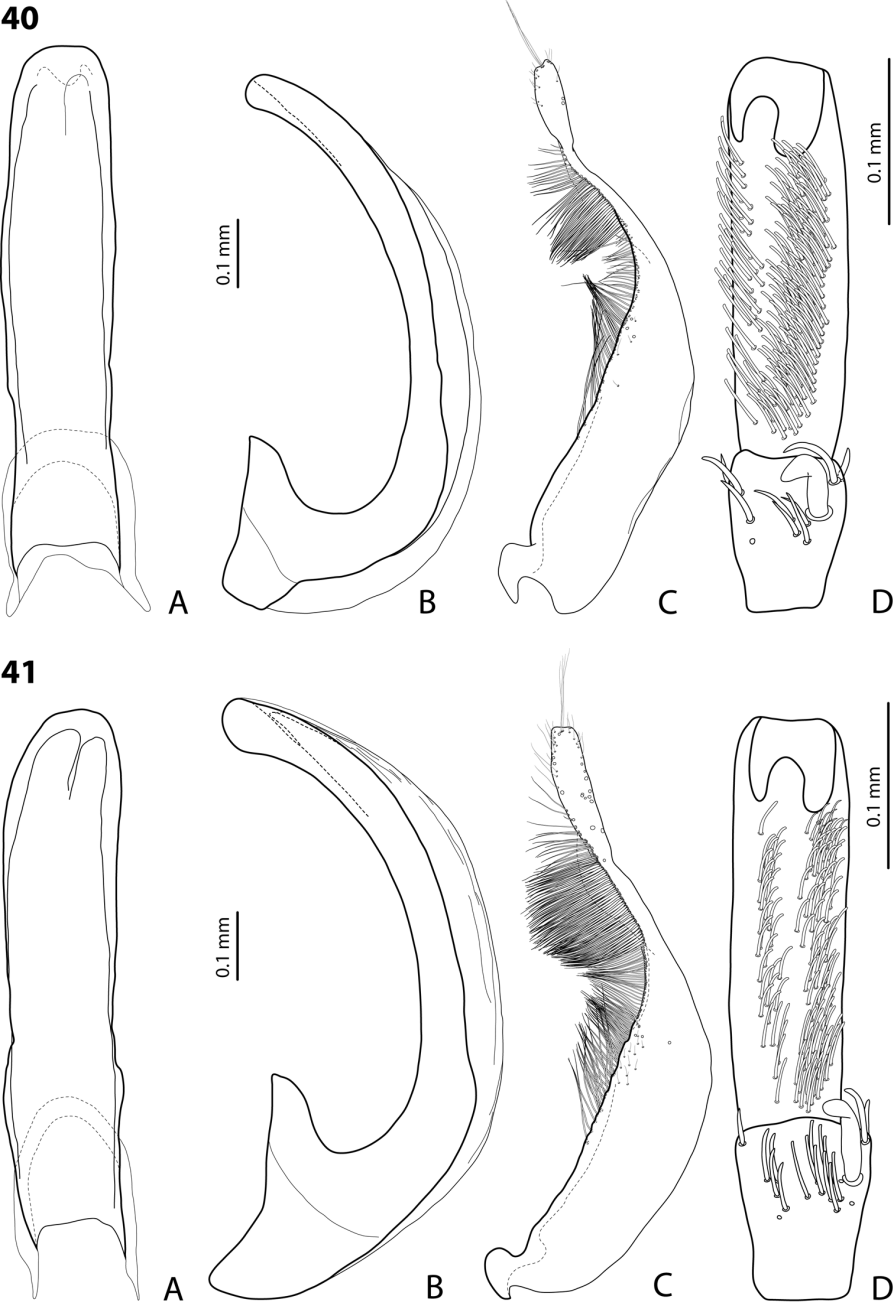
**40***Exocelinaheidiae* (Balke, 1998) **41***E.simbaijimi* sp. n. **A** Median lobe in ventral view **B** Median lobe in lateral view **C** Paramere in external view **D** Male protarsomeres 4–5 in ventral view.

**Figures 42–43.**
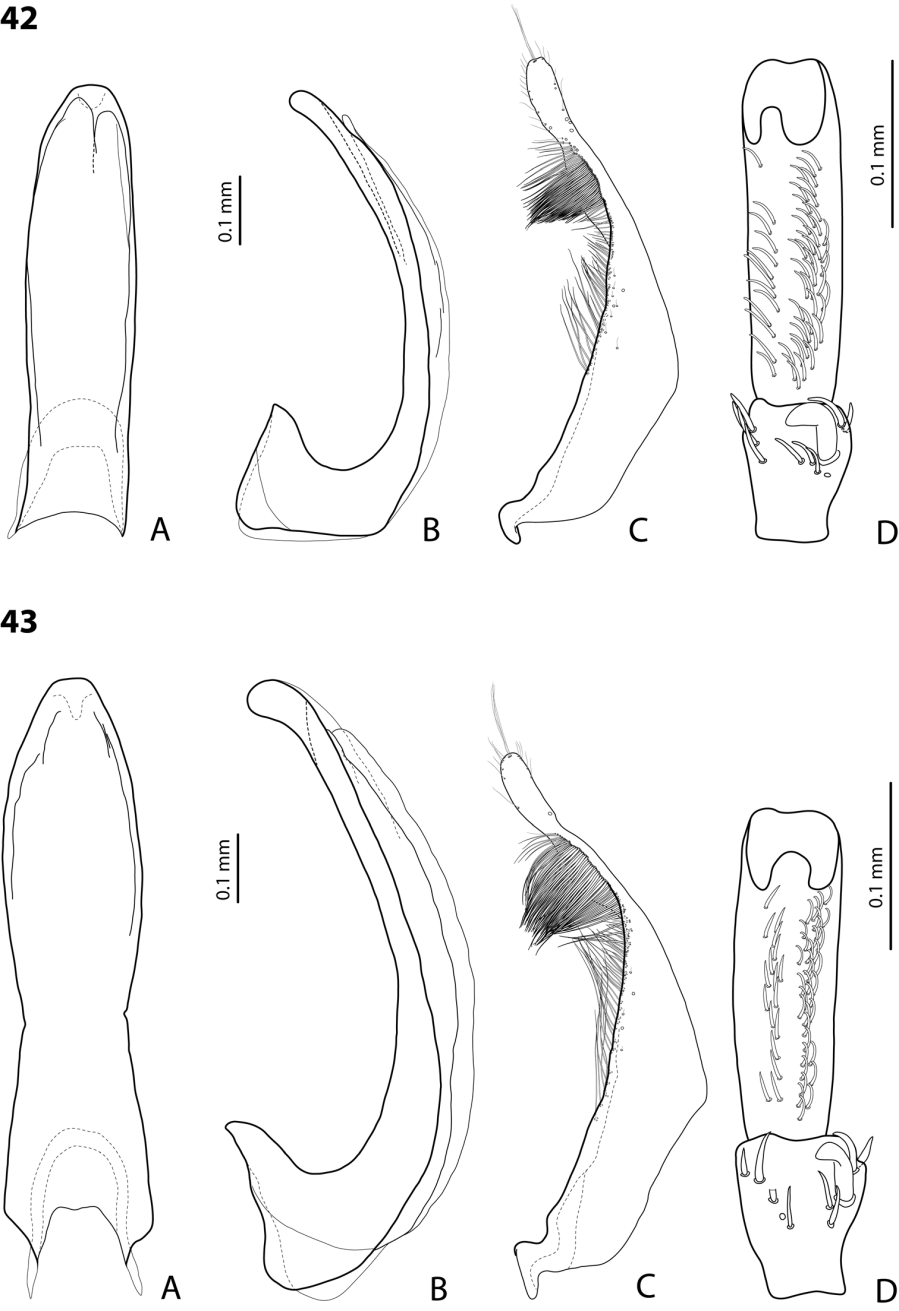
**42***Exocelinapseudopusilla* sp. n. **43***E.simbaiensis* sp. n. **A** Median lobe in ventral view **B** Median lobe in lateral view **C** Paramere in external view **D** Male protarsomeres 4–5 in ventral view.

**Figures 44–45.**
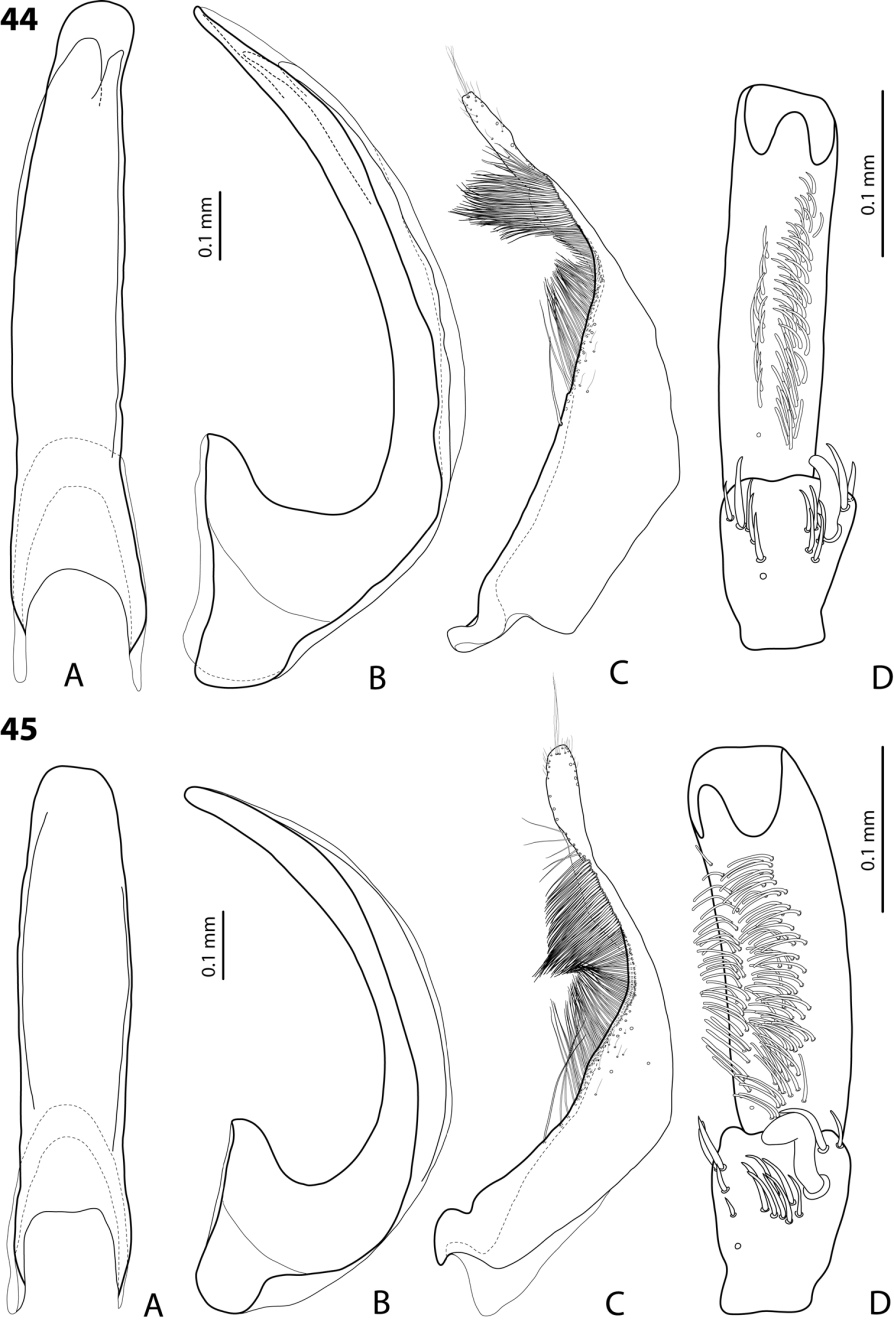
**44***Exocelinamenyamya* sp. n. **45***E.ambua* sp. n. **A** Median lobe in ventral view **B** Median lobe in lateral view **C** Paramere in external view **D** Male protarsomeres 4–5 in ventral view.

**Figures 46–47.**
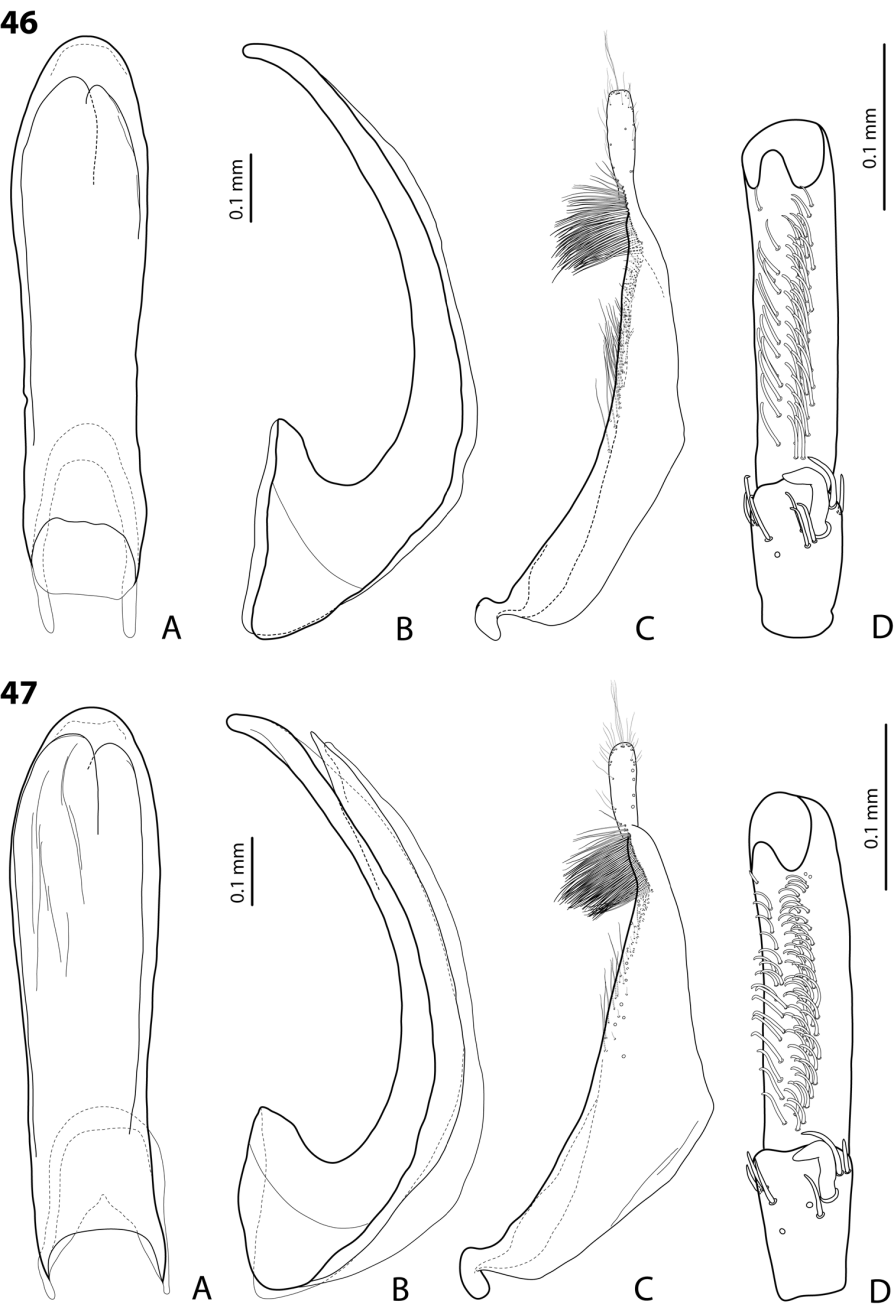
**46***Exocelinayoginofi* sp. n. **47***E.okapa* sp. n. **A** Median lobe in ventral view **B** Median lobe in lateral view **C** Paramere in external view **D** Male protarsomeres 4–5 in ventral view.

**Figure 48.**
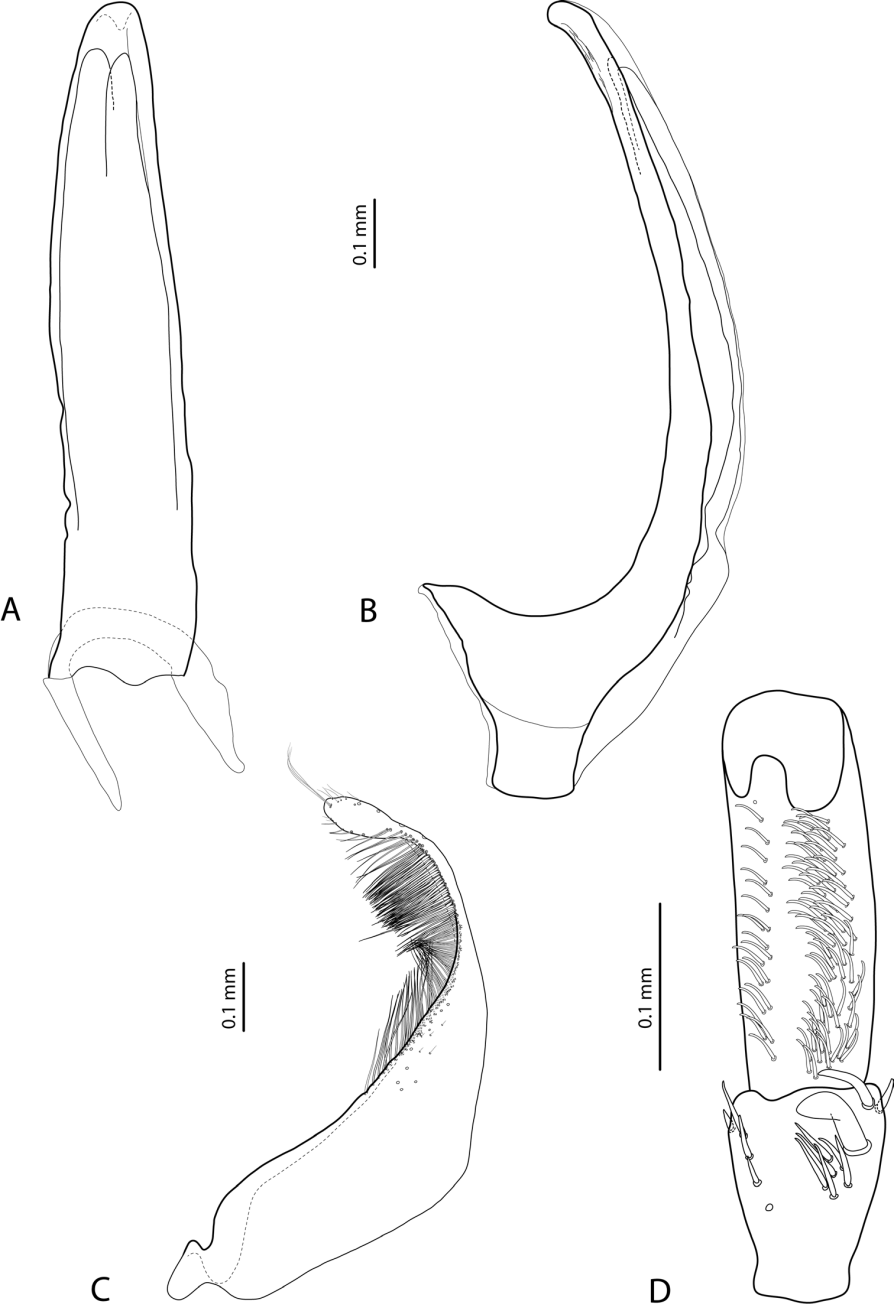
*Exocelinamendiensis* sp. n. **A** Median lobe in ventral view **B** Median lobe in lateral view **C** Paramere in external view **D** Male protarsomeres 4–5 in ventral view.

**Figure 49.**
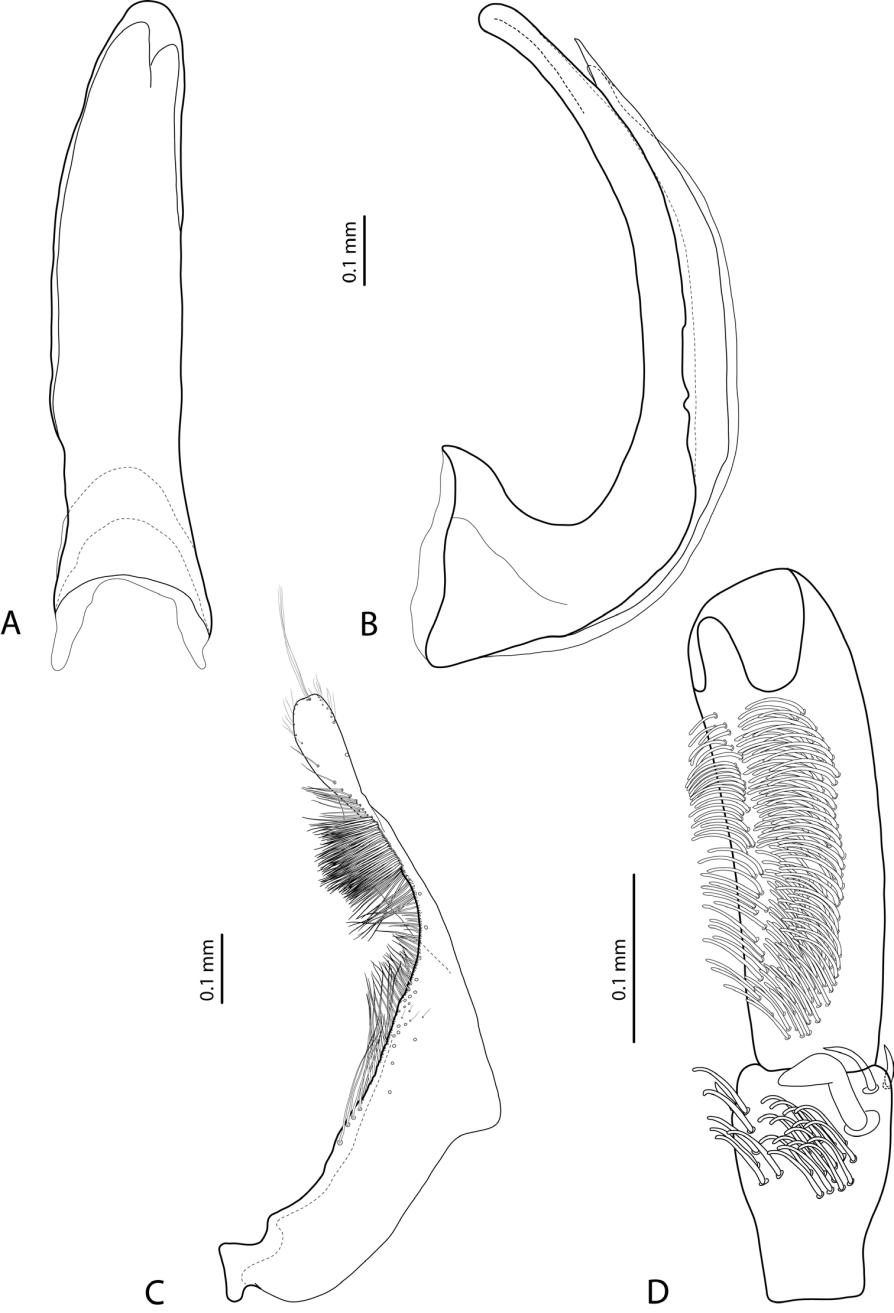
*Exocelinakumulensis* sp. n. **A** Median lobe in ventral view **B** Median lobe in lateral view **C** Paramere in external view **D** Male protarsomeres 4–5 in ventral view.

**Figure 50.**
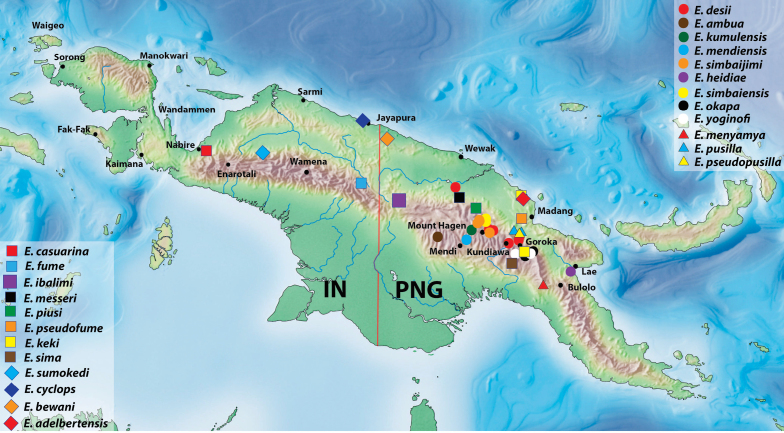
Map of New Guinea showing distribution of the species.

## Supplementary Material

XML Treatment for Exocelina adelbertensis

XML Treatment for Exocelina ambua

XML Treatment for Exocelina bewani

XML Treatment for Exocelina casuarina

XML Treatment for Exocelina cyclops

XML Treatment for Exocelina desii

XML Treatment for Exocelina fume

XML Treatment for Exocelina heidiae

XML Treatment for Exocelina ibalimi

XML Treatment for Exocelina keki

XML Treatment for Exocelina kumulensis

XML Treatment for Exocelina mendiensis

XML Treatment for Exocelina menyamya

XML Treatment for Exocelina messeri

XML Treatment for Exocelina okapa

XML Treatment for Exocelina piusi

XML Treatment for Exocelina pseudofume

XML Treatment for Exocelina pseudopusilla

XML Treatment for Exocelina pusilla

XML Treatment for Exocelina sima

XML Treatment for Exocelina simbaiensis

XML Treatment for Exocelina simbaijimi

XML Treatment for Exocelina sumokedi

XML Treatment for Exocelina yoginofi

## References

[B1] Balke M (1998) Revision of New Guinea *Copelatus* Erichson, 1832 (Insecta: Coleoptera: Dytiscidae): The running water species, Part I. Annalen des Naturhistorischen Museum Wien 100B: 301–341.

[B2] Balke M (1999) Two new species of the genus CopelatusErichson, 1832,subgenusPapuadytes Balke, 1998, from Papua New Guinea (Insecta: Coleoptera: Dytiscidae). Annalen des Naturhistorischen Museum Wien 101B: 273–276.

[B3] Balke M (2001) Replacement names for three New Guinea species of *Copelatus*, subgenus Papuadytes Balke, 1998 (Coleoptera: Dytiscidae). Annalen des Naturhistorischen Museum Wien 103B: 361–362.

[B4] Balke M Pons J Ribera I Sagata K Vogler AP (2007) Infrequent and unidirectional colonization of hyperdiverse *Papuadytes* diving beetles in New Caledonia and New Guinea. Molecular Phylogenetics and Evolution 42: 505–516. 10.1016/j.ympev.2006.07.019 16979911

[B5] Broun T (1886) Manual of the New Zealand Coleoptera. Parts III and IV. Wellington, Government Printer, 817–973.

[B6] Nilsson AN (2001) Dytiscidae. World Catalogue of Insects Vol. 3. Apollo Books, Stenstrup, 395 pp.

[B7] Nilsson AN (2007) *Exocelina* Broun, 1886, is the valid name of *Papuadytes* Balke, 1998. Latissimus 23: 33–34.

[B8] Nilsson AN Fery H (2006) World Catalogue of Dytiscidae—corrections and additions, 3 (Coleoptera: Dytiscidae). Koleopterologische Rundschau 76: 55–74.

[B9] Nilsson AN Hájek J (2018) A World Catalogue of the Family Dytiscidae, or the Diving Beetles (Coleoptera, Adephaga). Internet version 31.I.2018, http://waterbeetles.eu/documents/W_CAT_Dytiscidae_2017.pdf

[B10] Shaverdo HV Sagata K Balke M (2005) Five new species of the genus *Papuadytes* Balke, 1998 from New Guinea (Coleoptera: Dytiscidae). Aquatic Insects 27(4): 269–280. 10.1080/01650420500290169

[B11] Shaverdo HV Surbakti S Hendrich L Balke M (2012) Introduction of the *Exocelinaekari*-group with descriptions of 22 new species from New Guinea (Coleoptera, Dytiscidae, Copelatinae). ZooKeys 250: 1–76. 10.3897/zookeys.250.3715 PMC3558971 23378803

[B12] Shaverdo HV Hendrich L Balke M (2013) *Exocelinabaliem* sp. n., the only known pond species of New Guinea *Exocelina* Broun, 1886 (Coleoptera, Dytiscidae, Copelatinae). ZooKeys 304: 83–99. 10.3897/zookeys.304.4852 PMC3689123 23794909

[B13] Shaverdo HV Balke M (2014) *Exocelinakinibeli* sp.n. from Papua New Guinea, a new species of the *E.ullrichi*-group (Coleoptera: Dytiscidae). Koleopterologische Rundschau 84: 31–40.

[B14] Shaverdo H Sagata K Panjaitan R Menufandu H Balke M (2014) Description of 23 new species of the *Exocelinaekari*-group from New Guinea, with a key to all representatives of the group (Coleoptera, Dytiscidae, Copelatinae). ZooKeys 468: 1–83. 10.3897/zookeys.468.8506 PMC4296520 25610341

[B15] Shaverdo H Panjaitan R Balke M (2016a) A new, widely distributed species of the *Exocelinaekari*-group from West Papua (Coleoptera, Dytiscidae, Copelatinae). ZooKeys 554: 69–85. 10.3897/zookeys.554.6065 PMC4740830 26877680

[B16] Shaverdo H Sagata K Balke M (2016b) Description of two new species of the *Exocelinabroschii*-group from Papua New Guinea, with revision and key to all representatives of this species group (Coleoptera, Dytiscidae, Copelatinae). ZooKeys 577: 125–148. 10.3897/zookeys.577.7254 PMC4829886 27110191

[B17] Shaverdo H Sagata K Balke M (2016c) Taxonomic revision of New Guinea diving beetles of the *Exocelinadanae* group, with description of ten new species (Coleoptera, Dytiscidae, Copelatinae). ZooKeys 619: 45–102. 10.3897/zookeys.619.9951 PMC5090162 27829789

[B18] Shaverdo H Panjaitan R Balke M (2016d) *Exocelinaransikiensis* sp. nov. from the Bird’s Head of New Guinea (Coleoptera: Dytiscidae: Copelatinae). Acta Entomologica Musei Nationalis Pragae 56: 103–108.

[B19] Shaverdo H Wild M Sumoked B Balke M (2017) Six new species of the genus *Exocelina* Broun, 1886 from Wano Land, New Guinea (Coleoptera, Dytiscidae, Copelatinae). ZooKeys 665: 93–120. 10.3897/zookeys.665.11792 PMC5523169 28769629

[B20] Toussaint EFA Hall R Monaghan MT Sagata K Ibalim S Shaverdo HV Vogler AP Pons J Balke M (2014) The towering orogeny of New Guinea as a trigger for arthropod megadiversity. Nature Communications 5: 5: 4001. 10.1038/ncomms5001 24874774

[B21] Toussaint EFA Henrich L Shaverdo H Balke M (2015) Mosaic patterns of diversification dynamics following the colonization of Melanesian islands. Scientific Reports 5: 16016. 10.1038/srep16016 PMC4630634 26526041

